# Healing of ischemic injury in the retina

**DOI:** 10.1126/sciadv.adx7204

**Published:** 2026-01-21

**Authors:** Silke Becker, Zia L’Ecuyer Morison, Jordan Allen, Sama Saeid, Lee Sturgis, Austin Adderley, Ari Koskelainen, Frans Vinberg

**Affiliations:** ^1^ John A. Moran Eye Center, University of Utah, Salt Lake City, UT, USA.; ^2^ Department of Ophthalmology, UPMC Vision Institute, University of Pittsburgh, Pittsburgh, PA, USA.; ^3^ Interdepartmental PhD Neuroscience Program, University of Utah, Salt Lake City, UT, USA.; ^4^ Department of Biomedical Engineering, University of Utah, Salt Lake City, UT, USA.; ^5^ Department of Neuroscience and Biomedical Engineering, Aalto University, Espoo, Finland.; ^6^ Utah Lions Eye Bank, Salt Lake City, UT, USA.; ^7^ Brunson Center for Translational Vision Research, University of California, Irvine, CA, USA.

## Abstract

Neuro- and retinal degenerative diseases, including Alzheimer’s, stroke, age-related macular degeneration, and central retinal artery occlusion, rob millions of their independence. Studying these diseases in human retinas has been hindered by the rapid loss of neuronal activity after death. While some CNS activity has been restored postmortem, synchronized neuronal transmission beyond 30 min has remained elusive. We overcome this barrier by reviving and sustaining light signal transmission in human retinas recovered up to 4 hours after death and stored for up to 48 hours. We also introduce infrared-based ex vivo imaging for precise sampling, a closed perfusion system for drug testing, and an ex vivo ischemia-reperfusion model in mouse and human retina. This platform enables testing of neuroprotective and neurotoxic effects of drugs targeting oxidative stress and glutamate excitotoxicity. Our advances question the irreversibility of ischemic injury, support preclinical studies in vision restoration, offer insights into treating CNS ischemia, and pave the way for human donor eye transplantation.

## Teaser

Light signals in postmortem human retinas challenge that ischemic injury is irreversible and aid research to restore vision.

## INTRODUCTION

Until recently, consensus decreed that neurons in the central nervous system (CNS), including the retina, rapidly and irreversibly deteriorate after the blood circulation ceases (*1*–*3*). Recent discoveries challenge this long-held belief, with the pig brain regaining metabolic and spontaneous neuronal activity several hours after death, although synchronized global activity remains absent (*4*, *5*). However, efforts to preserve the brain have not exceeded 6 hours after circulation was restored, leaving the question of whether a more complete recovery might be possible over a longer timeframe.

Complimenting these observations, we recently restored postsynaptic ON-bipolar cell light responses in postmortem human retinas recovered within 20 min of circulation loss but not in eyes enucleated 1 to 5 hours postmortem (*6*). While inner retinal neurons can partially regain spontaneous activity and light responsiveness following brief hypoxia or ischemia (*7*–*10*), synchronized network activity of CNS tissues, including the retina, has not been achieved beyond 30 min after circulatory death. Partial recovery of inner retinal light responses several weeks after retinal ischemia in a rhesus monkey model of central retinal artery occlusion (CRAO) (*8*) suggests that retinal ischemic damage may be reversible with a sufficient recovery time.

Current treatments for CRAO center around rapidly restoring blood flow through thrombolysis, ocular massage, paracentesis, intraocular pressure reduction, or hyperbaric oxygen therapy (*11*–*13*). However, the narrow therapeutic time window and limited efficacy of these treatments result in legal blindness (20/200 visual acuity or worse) in the affected eye of 81% of patients with CRAO (*14*). Rapidly restoring blood flow is critical to effectively treat acute circulatory loss in the brain (and much more efficient than in CRAO), but attempts to prevent or repair ischemic damage to CNS neurons through neuroprotective strategies have so far failed. Extending the viability of neurons and understanding mechanisms that reverse ischemic damage could transform clinical outcomes, not only for patients with CRAO but also for the much larger population of patients with stroke.

In this study, we recover retinal function following prolonged ischemia. By maintaining retinal light responses in the laboratory, pioneering ex vivo infrared optical coherence tomography (OCT) and fundus imaging to precisely identify and sample retinal structures in human donor eyes, and engineering a small volume-closed perfusion system (*15*, *16*), we unlock the potential of the postmortem human retina as a powerful research tool. In addition, we present a functional ex vivo assay to model diseases associated with acute ischemia-reperfusion injury, such as CRAO, and to test drugs for ischemia-reperfusion injury.

## RESULTS

### Restoring and preserving light signaling in human donor retinas

We previously revived photoreceptor and ON-bipolar cell light responses when eyes were recovered within 20 min of circulatory arrest (cross-clamp). In contrast, we recorded only photoreceptor light responses if enucleation was delayed by more than 1 hour (*6*). To explore the necessary time window for restoring in vivo–like light responses with ex vivo electroretinography (ERG), we partnered with the Utah Lions Eye Bank and the Organ Procurement Organization DonorConnect in Salt Lake City, Utah, to obtain whole human globes or posterior poles (after collection of the corneas for transplantation) from donations after brain and cardiac death (DBD and DCD, respectively) with informed consent from the donors’ families.

**Fig. 1.**
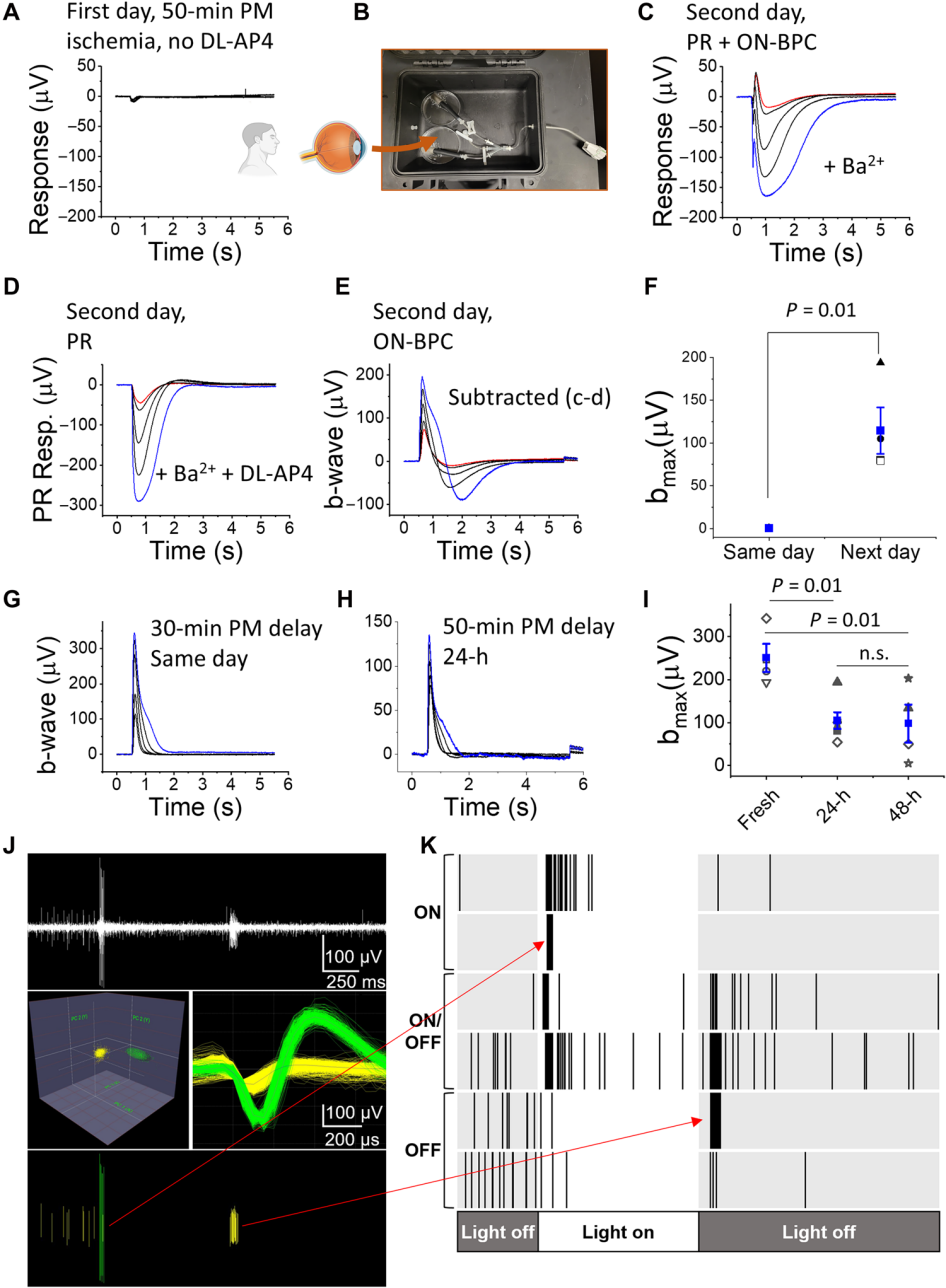
Restoring and preserving ERG b-waves and ganglion cell light responses in organ donor eyes after overnight eyecup incubation. (**A**) Examples of ex vivo ERG light responses from the peripheral retina of an eye enucleated 50-min postmortem, recorded on the same day. In the absence of DL-AP4 only small a-waves were observed. (**B**) Overnight incubation of the eyecup in oxygenated media in darkness. (**C**) Examples of light responses of a peripheral retina sample (after overnight eyecup incubation) in the presence of Ba^2+^ alone and (**D**) combined with DL-AP4. (**E**) Subtracting responses in (D) from those in (C) isolates a strong b-wave. (**F**) Maximum b-wave amplitudes [i.e., blue trace in E from three donors with 50- to 65-min enucleation delay, recorded ~3 hours (h) postmortem (same day) or after overnight incubation (next day)] (blue: mean ± SE). (**G**) Ex vivo ERG b-waves determined by subtraction, as shown in (C) to (E), from human DBD eye enucleated within 30 min after death and recorded on the same day and (**H**) from human DCD eye enucleated within 50 min after death and recorded following 48-hour incubation. (**I**) Maximal b-wave amplitudes (mean ± SE) from peripheral samples recorded soon after enucleation (fresh, three donors with <30-min enucleation delay), 24 (five donors with <60-min enucleation delay), and 48 hours (four donors with <60-min enucleation delay) after enucleation. Light flash strengths ranged from 19 to 1014 photons (530 nm) μm^−2^. (**J**) (Top) High-pass filtered multielectrode array recording showing distinct ON and OFF RGC responses in peripheral retina from an eye with a 70-min enucleation delay and after 24-hour incubation. Stimulus luminance was 24 cd.s/m^2^. Waveforms were clustered on the basis of principle components (middle) and spikes were isolated (bottom). Spikes were used to generate raster plots (red arrows) with two examples cells each of ON, ON/OFF, and OFF RGC light responses (**K**).

Peripheral retinas from five DBD or DCD donors, collected 50 to 60 min after circulatory arrest, produced only small photoreceptor light responses during the first 1 to 2 hours after arriving in the laboratory (Fig. 1A). Since ischemic damage can be reversed in models of CRAO (*8*), we tested whether overnight incubation could improve light responses. By incubating eyecups (with the cornea, lens, and some of the vitreous removed) or pieces of the posterior eye in oxygenated bicarbonate-buffered Ames’ media at room temperature overnight (Fig. 1B), we revived postsynaptic ON-bipolar cell light responses (Fig. 1, C, E, and F, in the presence of Ba^2+^, which blocks K^+^ currents in Müller glia) and recorded large photoreceptor light responses (Fig. 1D) in the additional presence of DL-AP4, which silences ON-bipolar cell light responses by continuously activating mGluR6 receptors in eyes collected within 50 to 60 min. Notably, this protocol also restored ON-bipolar cell responses in two of five pairs of research donor eyes enucleated 3 to 4 hours postmortem (fig. S1).

Maintaining the attachment between the retina and retinal pigment epithelium (RPE) is essential: Isolated mouse retinas lost almost all of their ON-bipolar cell and much of their photoreceptor light responses during overnight incubation, even in oxygenated media (fig. S2). Thus, postmortem ischemic injury can heal, provided the eyes are enucleated and placed in oxygenated media within approximately 1 hour, or in some cases, several hours, from the loss of circulation, and the retina remains attached to the RPE.

Although human retinal organoids and, on occasion, neural retinas have been cultured long term (*17*–*25*), functional preservation of the whole human posterior eye ex vivo remains largely unexplored. To determine the duration for which retinal light responses can be maintained, we incubated posterior eyecups in oxygenated bicarbonate-buffered Ames’ media at room temperature, as described above (Fig. 1B). We punched 5-mm retina samples near the macula at 1 to 3 (fresh), 24, and 48 hours postmortem and determined the subtracted ex vivo ERG b-wave amplitude (ON-bipolar cell response) as a sensitive biomarker for viability, as described in Fig. 1, C to E. These ON-bipolar cell responses were robust for up to 48 hours, although the maximum response amplitudes declined over time (Fig. 1I).

Retinal output neurons, called retinal ganglion cells (RGCs), carry the light signal to the brain for visual perception. Thus, restoring and preserving their light responses will be critical for many applications, such as whole-eye transplantation. It is known that damage to the optic nerve initiates pathways that lead to RGC death in vivo (*26*). It is unknown, however, how long human RGCs remain light-responsive ex vivo after optic nerve transection. Here, we measured light responses of RGCs in peripheral human retina samples obtained from one DCD donor (ARPAH6) eye that was enucleated 70 min postmortem and preserved as an eyecup for 24 hours under the conditions described above (see Fig. 1). We were able to record light responses from multiple different RGC types (Fig. 1, J and K, and fig. S3), indicating that RGC function can be restored and preserved in the whole eyecup even after more than 1 hour of warm ischemia time and at least up to 24 hours after optic nerve transection.

### Visualizing and collecting human retina samples for ex vivo ERG recordings

The macula and fovea, located in the central retina and unique to humans and nonhuman primates among mammals, mediate high-acuity daytime color vision (*27*). Current animal models inadequately mimic the physiology and pathophysiology of the central retina, limiting progress in studying diseases that affect high-acuity vision, e.g., age-related macular degeneration (*28*). Existing technologies do not permit studying macular physiology in postmortem eyes from healthy donors and patients with retinal diseases. To fill this gap, we adapted an existing imaging platform to precisely collect samples from human donor eyes while preserving retinal light sensitivity.

**Fig. 2.**
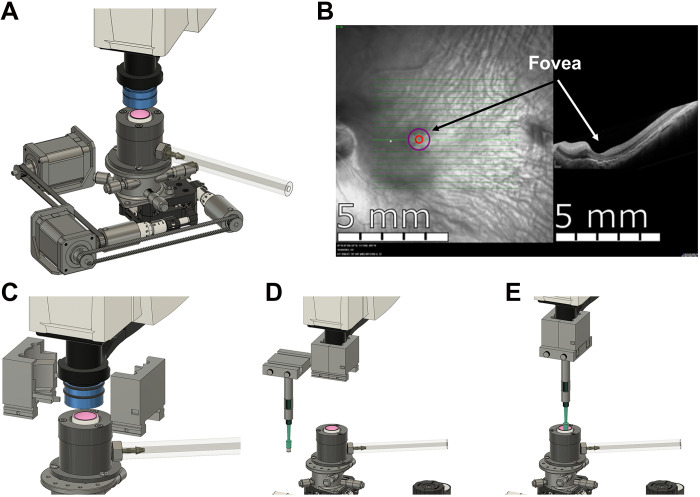
Foveal analysis and procurement automation. (**A**) Fundus and OCT imaging of ex vivo human eyecup. (**B**) Deep learning models scan the retina, locate the fovea and move the retina into position. (**C** and **D**) Biopsy punch attachment affixed to OCT lens. (**E**) Sampling of the fovea.

We converted a Heidelberg Spectralis OCT and fundus imaging platform for ex vivo analysis of eyecups under infrared light (Fig. 2, A and B). Typically used for high-resolution cross-sectional images of the retina in clinical settings, we adapted the OCT to image postmortem eyecups filled with Ames’ media after the cornea and lens had been removed to provide a clear optical path. A custom scanner lens adapter was created with Fusion 360, with which we securely fitted an additional lens to correct refraction after removing the lens and cornea (shown in blue in Fig. 2, A and C; noncontact animal imaging lens, +25 Diopter, from Heidelberg Engineering Inc.). This setup allows us to image the retina and accurately locate the fovea in human donor eyes (Fig. 2B).

A punch mechanism attached to a sliding lock to collect retinal samples (Fig. 2, C to E) achieved a puncture localization accuracy of 0.100 ± 0.066 mm across 24 trials (fig. S4A). Although effective with a 100% success rate (nine of nine) under illuminated conditions (fig. S5), this method was prone to human error under dim red light in our electrophysiology suite, prompting us to develop two deep learning models that automatically locate the fovea. The first model used a binary classification scheme to determine whether OCT images contain a fovea with 98% accuracy in the training and 100% accuracy in the validation phases (fig. S4B). The second model, based on the Ultralytics YOLO v8 architecture, precisely located the foveal coordinates with 97% precision and a 90% recall across images. This model effectively identified instances of the target class, “fovea,” in 39 validation images (fig. S4C). All code, artificial intelligence (AI) models, designs, and training data are available on Zenodo (https://zenodo.org/records/16014189).

**Fig. 3.**
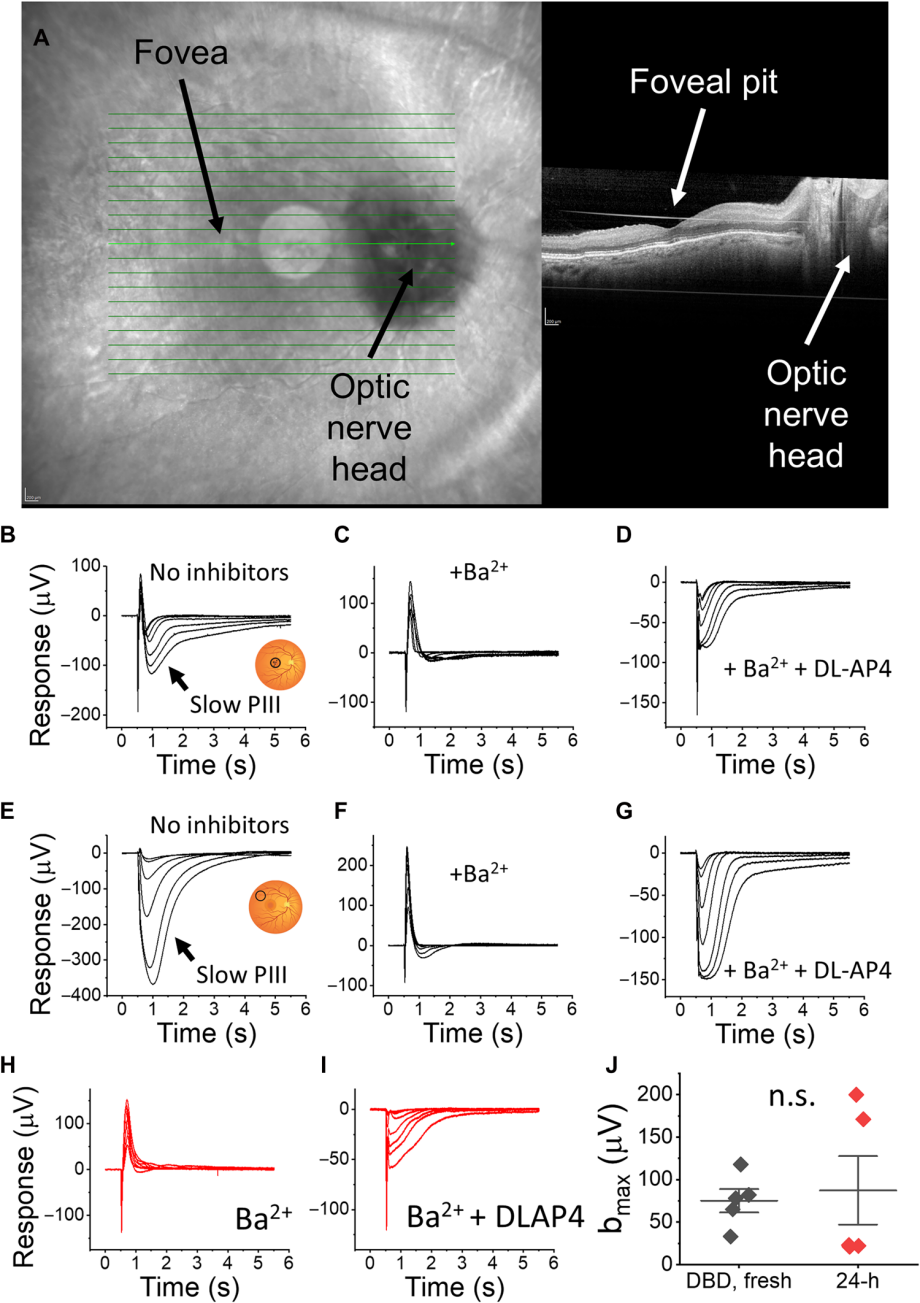
Imaging and recording of light responses from the central and peripheral human retina. (**A**) Infrared fundus and OCT image of the postmortem human retina from an eye enucleated 30 min postmortem. Retinal light responses in the central (**B** to **D**) and peripheral (**E** to **G**) retina from a DBD eye. Macular and peripheral light responses from the same eye shown in (A), (B) and (E) without inhibitors, (C) and (F) with 100 μM BaCl_2_ to block glial responses, and (D) and (G) with BaCl_2_ and 40 μM DL-AP4 to remove glial and inner retina components. (**H** and **I**) Macular light responses under same recording condition from a sample that was punched from the eyecup that was incubated for 24 hours as described in Fig. 1. Light flash strengths at 530 nm wavelength ranged from 19 to 3525 photons μm^−2^. (**J**) Maximum b-wave amplitudes determined as shown in Fig. 1 for control maculae that were obtained from DBD eyes recovered within 30 min and recorded within few hours from the cross-clamp [black diamonds; donors 7, 8, 9, and 11 from Abbas *et al.* (*6*) and UFT5 from this study] and for maculae that were obtained from DBD or DCD eyes recovered within 40 to 70 min and recorded 24 hours from cross-clamp (DNB) or time of death (DCD) (red diamonds; donors ARPAH1, 6, 7, 9, and 10).

Using the described fundus and OCT imaging, we collected the foveal pits and surrounding maculae (Fig. 3A). Retinas from a DBD donor (who had been on life support for 4 days and whose eyes had been enucleated 30 min after cross-clamp) showed strong photoreceptor and downstream retinal neuronal light responses in the central and peripheral retina regions (Fig. 3, D, C, G, and F, respectively). This is consistent with our previous work, which revived robust outer and inner retina light responses with postmortem enucleation delays of 0 to 20 min (*6*).

In addition, we identified a Ba^2+^-sensitive slow negative wave, known as the slow PIII (glial) component, in the macular region of the human retina (Fig. 3B). The slow PIII wave has been extensively described in cold-blooded species and rodents and likely arises from K^+^ buffering by Müller glial cells (*29*–*32*). In the human peripheral retina, the slow PIII component was noticeably larger and similar to that in mice, compared to the smaller Ba^2+^-sensitive component in the macula (Fig. 3, B and E).

Our data above (Fig. 1) demonstrated revival and preservation of light responses after ~1 hour of warm ischemia and 48 hours of incubation of the posterior globes when nutrients and oxygen were delivered from the vitreous space. The human central retina has higher metabolic activity compared to that of the peripheral retina (*33*) and could be more sensitive to ischemia or more vulnerable to loss of light responses in the ex vivo environment. To address this question, we recorded ex vivo ERG light responses from the maculae from five DBD or DCD donor eyes that were enucleated 40 to 70 min after cross-clamp (DBDs) or time of death (DCDs) and incubated as eyecups for 24 hours as described in Fig. 1. Light responses were recorded in Ames’ media (Fig. 3H) and in Ames’ media supplemented with DL-AP4 to block inner retinal signals (Fig. 3I), allowing us to derive the ON-bipolar cell response (*b*_*max*_) by subtraction as described in Fig. 1. Despite some variability, we were able to record b-wave responses after the 24-hour incubation period (Fig. 3J, red diamonds) from all of the five donors. In some cases, responses were even larger than in fresh controls, likely reflecting the same healing process we described for the human peripheral retina above.

### Drug testing in the human retina and development of an ischemia-reperfusion injury model

**Fig. 4.**
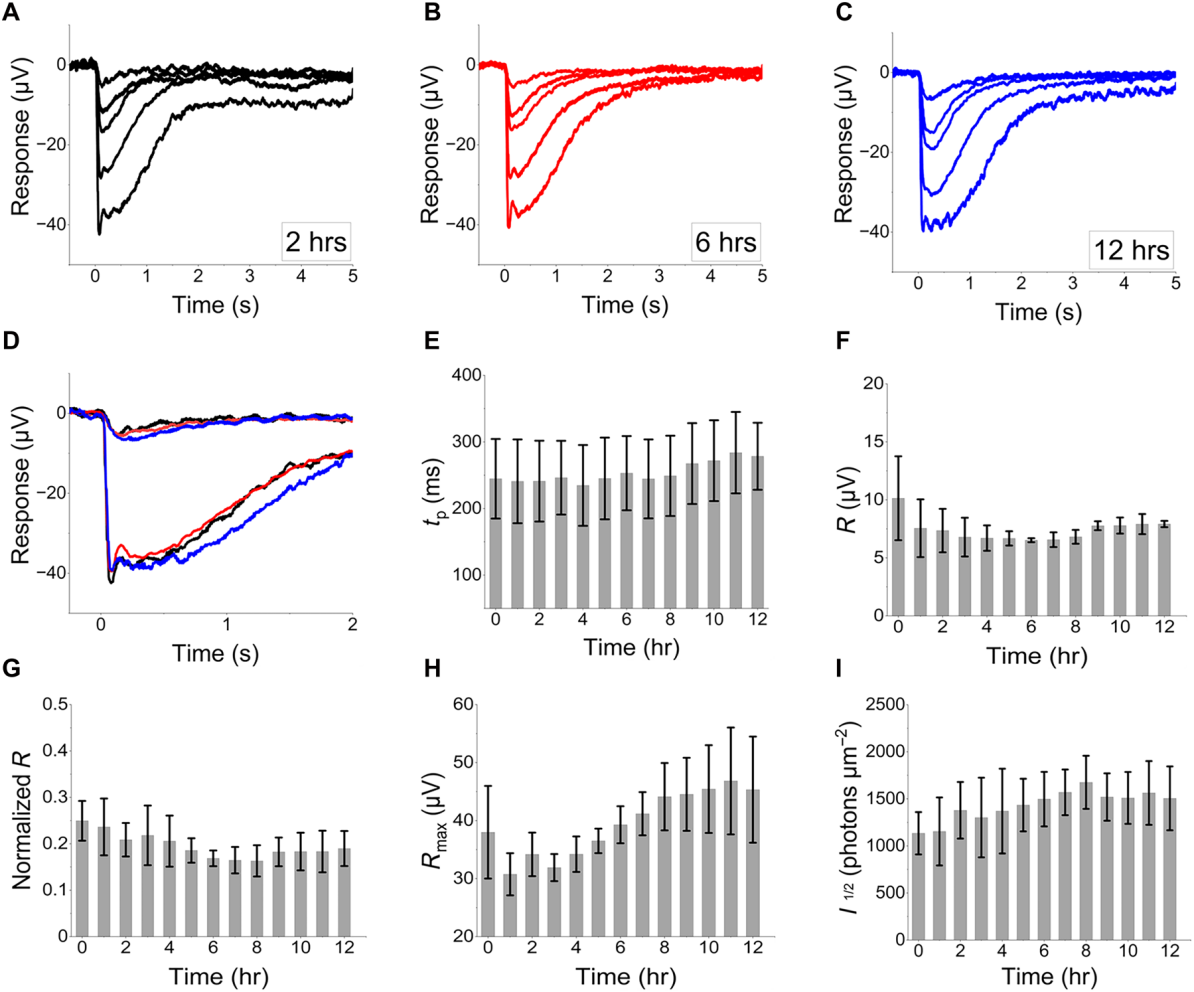
Preservation of ERG a-waves in the human retina during small-volume closed perfusion. (**A** to **C**) Example traces from a 38-year-old anoxic brain injury case with a one-hour post-mortem enucleation delay. (**D**) Preservation of dim and bright photoreceptors light responses at 2 (black), 6 (red), and 12 hours (hrs; blue) after perfusion was started. (**E**) Time to peak, (**F**) dim stimulus amplitude (R), (**G**) normalized amplitude to the maximal response, (**H**) maximum response and (**I**) light sensitivity (*I*_1/2_). Data in (E) to (I) are from three donors (UFT8, D54_2, and D55, see table S1), mean ± SEM.

To create a drug testing platform that minimizes drug usage (*15*, *16*), we built a closed perfusion system that recirculates small perfusate volumes (150 to 180 ml), as recently described (*15*). After overnight incubation of three human donor eyecups with 1- to 4-hour enucleation delays, peripheral retina samples maintained stable ex vivo rod photoreceptor light responses for at least 12 hours after initiating perfusion in the ex vivo ERG specimen holder. During this time, the dim flash response time-to-peak and amplitude, the normalized amplitude to the maximal response, the maximum response, the normalized maximum response to the initial response, and the flash strength producing half-maximal responses remained stable (Fig. 4). Consistent results from all three donor retinas confirmed that our setup preserves retinal function during extended recordings with a minimal perfusate volume.

### Acute oxygen deprivation in the mouse and human retina as a model for ischemia/reperfusion injury

Several in vivo animal models study ischemia-reperfusion injury in the eye and brain caused by, e.g., cardiac arrest, stroke, and central retinal artery and vein occlusion (*8*, *34*–*37*). However, no ex vivo models exist to assess retinal function following ischemia-reperfusion injury in postmortem human retinas. To address this gap and expand the use of our ex vivo testing platform, we developed an acute ischemia-reperfusion injury model that replicates neuronal damage caused by hypoxia and reoxygenation. This model is based on hypoxia/reoxygenation since our previous work demonstrated that postmortem loss of retinal light responses is likely caused by hypoxia, not tissue acidification due to ischemia (*6*).

To determine the relative susceptibility of different retinal neuronal cell types to hypoxia, we superfused isolated mouse retinas with hypoxic Ames’ media (~2.5% O_2_) while recording ex vivo ERG. We observed that ON-bipolar cells lost their light responses within several minutes, while photoreceptors retained >50% of theirs for at least 30 min (fig. S6).

**Fig. 5.**
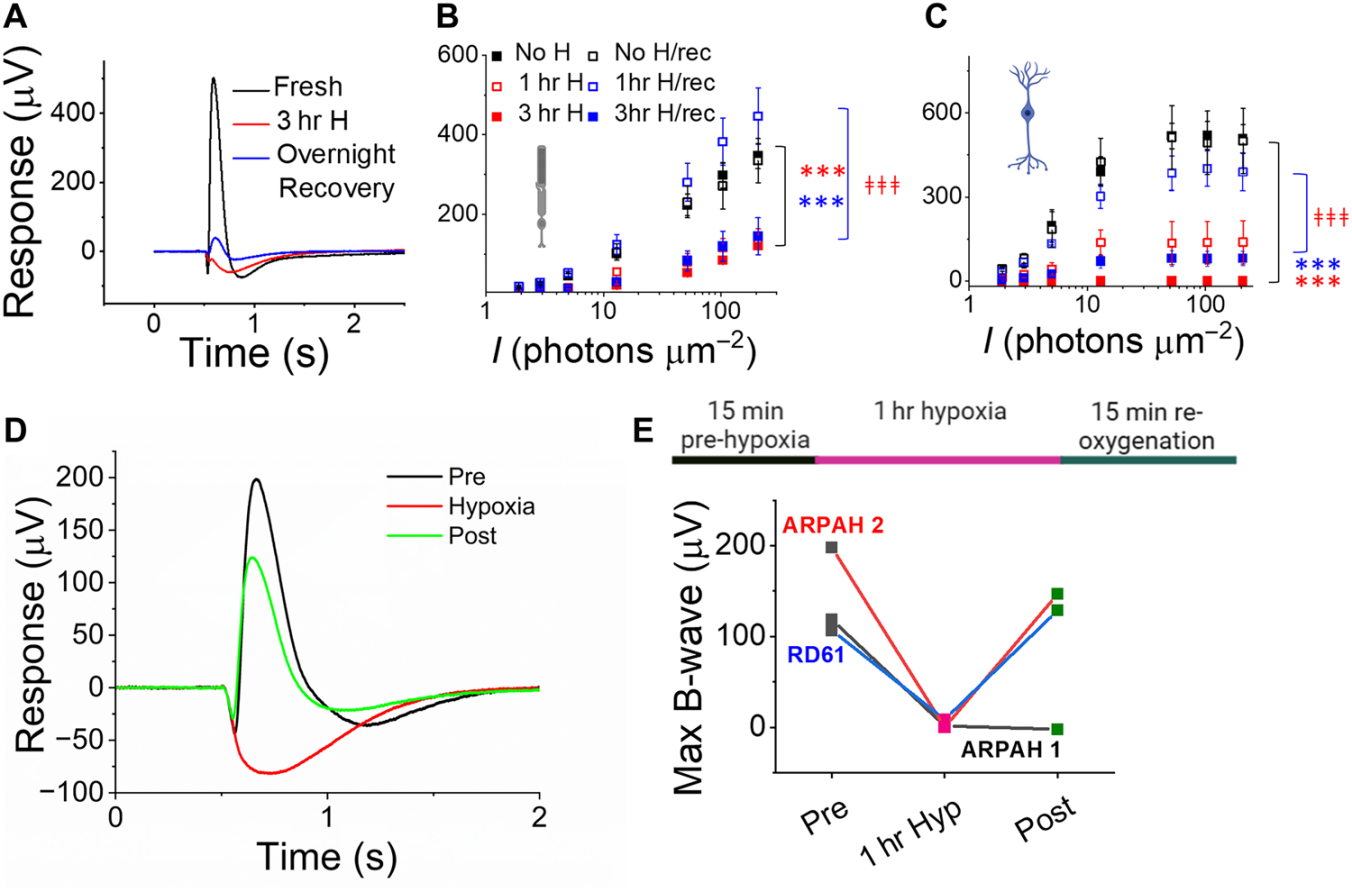
Ischemia-reperfusion injury model in the mouse and human retina. (**A**) Example traces showing rod photoreceptor light responses in *Gnat2* knockout mouse retinas immediately after death (black), after 3 hours (hr) of hypoxia (red), and after 3 hours of hypoxia followed by overnight recovery in oxygenated media (blue). (**B**) Averaged *Gnat2* knockout mouse rod photoreceptor and (**C**) ON-bipolar cell responses after 1 or 3 hours of hypoxia with and without overnight recovery in oxygenated media compared to retinas kept in oxygenated media without hypoxia (*n* = 2 to 6). Two-way ANOVA followed by Bonferroni post hoc test, ***P < 0.001 (compared to oxygenated control) and ‡‡‡*P* < 0.001 (compared to hypoxia). (**D**) Example traces showing light responses in human donor retina in oxygenated media (black), after perfusion with hypoxic media in the ex vivo specimen holder for 1 hour (red), and after recovery in oxygenated media for 15 min (blue). (**E**) ON-bipolar cell light responses from three human donor retinas under the same conditions as in (D).

We determined the time course of ischemia/reperfusion injury by measuring light responses in mouse eyecups exposed to low oxygen concentrations: Light responses were substantially reduced immediately after 1 and 3 hours of hypoxia, with longer hypoxia resulting in greater loss of ON-bipolar cell light responses (Fig. 5, A to C). Overnight incubation in oxygenated Ames’ media fully restored photoreceptor and ON-bipolar cell function after 1 hour of hypoxia, but only partially restored function after 3 hours of hypoxia (Fig. 5, A to C). When we superfused the retina in the specimen holder with hypoxic Ames’ media for 1 hour (by bubbling the perfusion media with 95% N_2_ and 5% CO_2_) and simultaneously recorded light responses, ON-bipolar cell light responses were eliminated (Fig. 5E). However, in two out of three samples, these responses substantially recovered within 15 min of reoxygenation (Fig. 5, D and E).

### Mechanisms of ischemic damage and repair

**Fig. 6.**
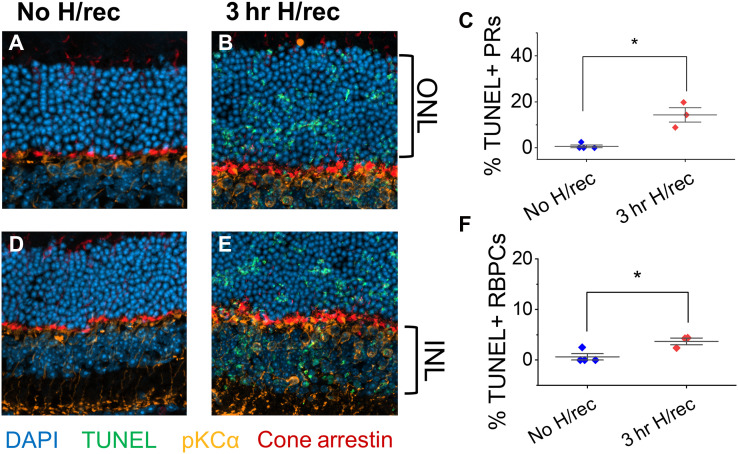
Limited cell death during hypoxia and recovery. TUNEL-positive photoreceptors (PRs) in the outer nuclear layer (ONL, **A** and **B**) and rod bipolar cells (RBPCs) in the inner nuclear layer (INL, **D** and **E**) of mouse retinas kept in oxygenated media [(A) and (D)] or treated with 3 hours (hr) of hypoxia (H) followed by overnight recovery (rec). Retinal sections were costained with the nuclear marker DAPI, the RBPC marker PKCa, and cone arrestin. (**C**) and (**F**) depict the proportion of TUNEL-positive cells of the total number of DAPI-stained cells in the ONL and INL. *n* = 3 to 4 mice.

We hypothesized that the incomplete functional recovery of light responses after 3 hours of hypoxia is due to the induction of cell death pathways. We found that 3 hours of hypoxia followed by overnight recovery increased the number of terminal deoxynucleotidyl transferase–mediated (TUNEL)–positive photoreceptors in the outer nuclear layer and of protein kinase Cα (PKCα)– and TUNEL-positive rod bipolar cells in the inner nuclear layers of mouse retinas (Fig. 6). However, the extent of TUNEL-positive cells (<20% of photoreceptors and <5% of rod bipolar cells) did not correlate with the much greater degree of functional loss observed (Fig. 5). We also stained retinas for glial fibrillary acidic protein (GFAP) and Iba1 to assess Müller glia and microglia (fig. S7). GFAP staining remained consistent with expression in astrocytes, with no evidence of Müller cell gliosis. Microglia remained in the inner and outer plexiform layers, but showed signs of changing morphology, indicative of subtle microglia activation, after overnight incubation in oxygenated media (with or without preceding hypoxia).

Ischemia-reperfusion injury causes multi-factorial damage before cell death, including an oxidative stress response from reduced form of nicotinamide adenine dinucleotide phosphate (NADPH) oxidase and the mitochondrial electron transport chain. Reduced adenosine 5′-triphosphate production during hypoxia impairs intracellular Ca^2+^ extrusion, and increased intracellular Ca^2+^ activates NADPH oxidase (*38*). The primary reactive oxygen species (ROS) produced by NADPH oxidase, superoxide and H_2_O_2_, open mitochondrial permeability transition pores, uncouple the mitochondrial electron transport chain, and further increase superoxide production (*39*). In addition, succinate accumulates, e.g., in the ischemic heart (*40*), which after reperfusion generates ROS by reversing complex I of the mitochondrial electron transport chain.

**Fig. 7.**
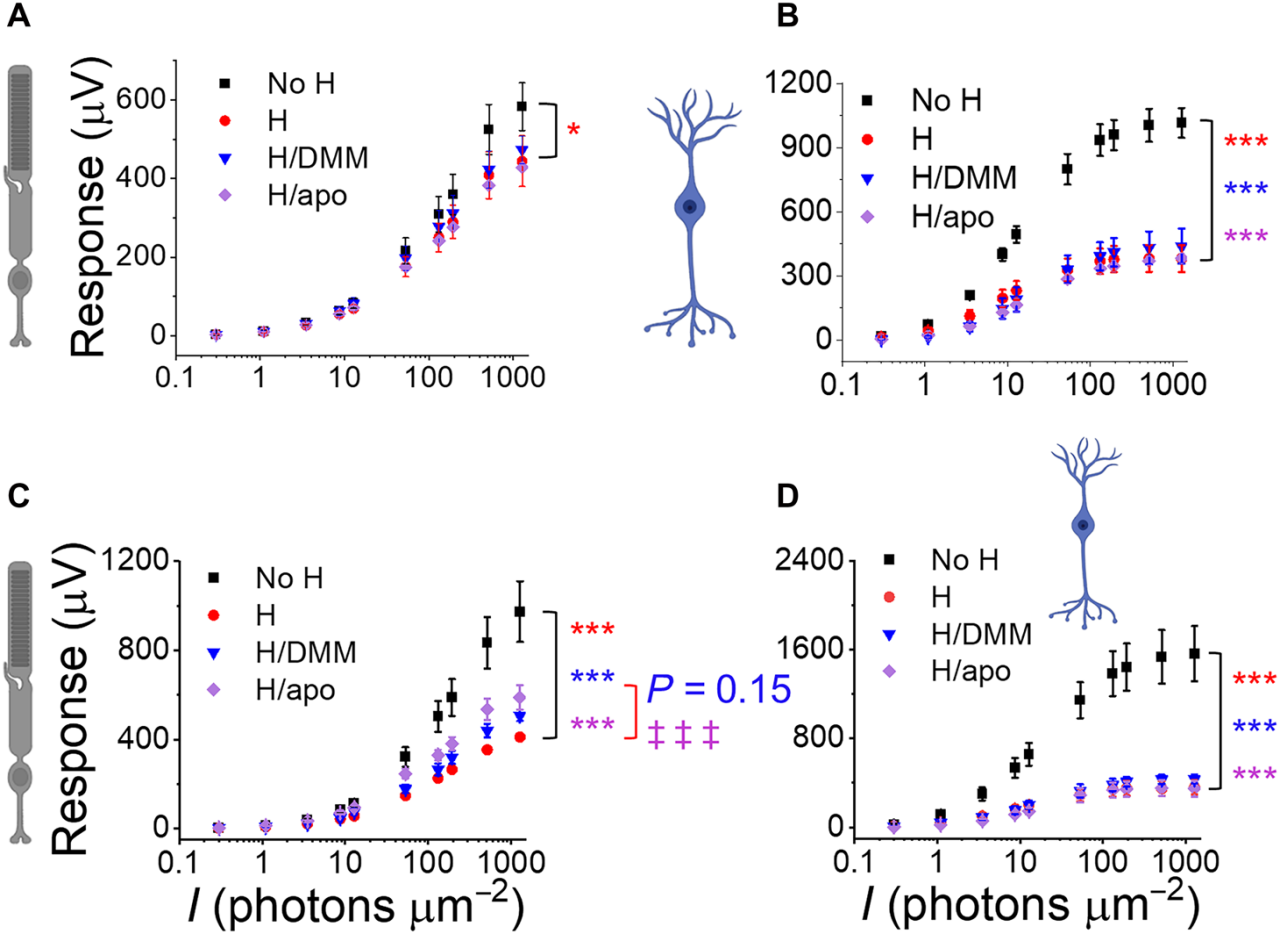
Preventing retinal injury during hypoxia and reoxygenation. Photoreceptor (**A** and **C**) and ON-bipolar cell light responses (**B** and **D**) in oxygenated media or [(A) and (B)] immediately after 1 hour of hypoxia (H) or [(C) and (D)] after 3 hours of hypoxia (H) with overnight incubation in oxygenated Ames’ media in the absence or presence of dimethyl malonate (DMM, 10 mM) or apocynin (apo, 400 mM, *n* = 4 to 6). Two-way ANOVA followed by Bonferroni post hoc test, *P < 0.05, ***P < 0.001 (compared to oxygenated control), and ‡‡‡*P* < 0.001 (compared to hypoxia).

To counteract this damage, we tested pharmacological agents: Dimethyl malonate (DMM; 10 mM), a succinate dehydrogenase blocker that prevents succinate accumulation during hypoxia and reduces ROS production in the mitochondrial electron transport chain during reoxygenation (*41*), and apocynin (400 μM), a blocker of NADPH oxidase assembly (*42*). Neither drug prevented the immediate loss of retinal light responses after 1 hour of hypoxia (Fig. 7, A and B). However, apocynin improved overnight recovery of photoreceptor, but not ON-bipolar cell, function after 3 hours of hypoxia (Fig. 7, C and D). DMM showed a nonsignificant trend toward improved photoreceptor function (Fig. 7, C and D). This implies that excessive ROS production during reoxygenation impairs photoreceptor but not ON-bipolar cell responses.

By carefully timing and combining these inhibitors, we revealed more of the underlying repair mechanisms: DMM most effectively protected photoreceptors when administered during hypoxia or just before reoxygenation (Fig. 7C and fig. S8A), suggesting that succinate accumulation during hypoxia leads to a detrimental burst of ROS by reversing succinate dehydrogenase immediately upon reoxygenation. Applying DMM only during reoxygenation did not protect photoreceptor function (fig. S8A).

ROS may not play a purely detrimental role in hypoxic damage and its repair; a basal amount of ROS may be protective: Combining DMM and apocynin was less effective in photoreceptors than apocynin alone (fig. S8C). The general ROS scavenger α-lipoic acid (1 mM) impaired photoreceptor recovery, while 0.1 mM α-lipoic acid did not affect recovery after hypoxia (fig. S8C). None of these pharmacological agents alone or in combination was protective for ON-bipolar cells (fig. S8, B and D). These findings suggest that excessive ROS production by the electron transport chain and the NADPH oxidase may not be the main mechanisms of damage to ON-bipolar cells, but likely damages photoreceptors. Limited ROS production may be necessary to prevent or repair ischemia-reperfusion injury in both cell types.

**Fig. 8.**
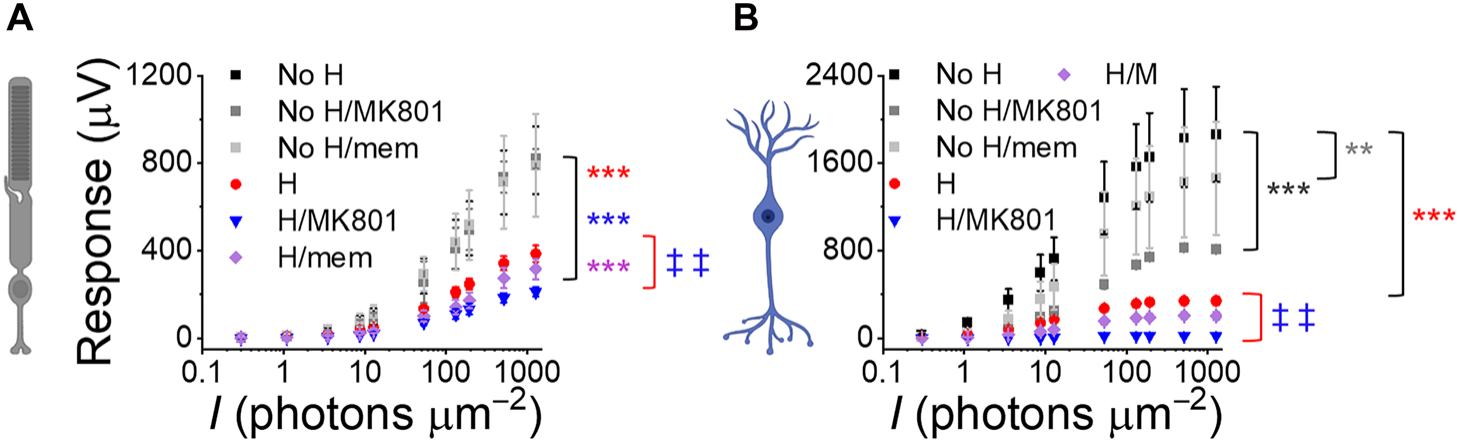
NMDA receptor blockers do not prevent retinal injury during hypoxia and reoxygenation. Photoreceptor (**A**) and ON-bipolar cell light responses (**B**) in oxygenated media (no H) in the absence or presence of MK-801 (300 μM) or memantine (mem, 100 μM) or after 3 hours of hypoxia (H) with overnight incubation in oxygenated Ames’ media in the absence or presence of MK-801 or memantine (*n* = 2 to 6). Two-way ANOVA followed by Bonferroni post hoc test, **P < 0.01, ***P < 0.001 (compared to oxygenated control), and ‡‡*P* < 0.01 (compared to hypoxia).

Last, we tested two NMDA receptor blockers, MK-801 (300 μM) and memantine (100 μM), to prevent excitotoxicity in inner retinal neurons caused by excessive glutamate release from damaged photoreceptors and ON-bipolar cells under hypoxic conditions and during reoxygenation. Neither drug was protective and MK-801 was detrimental to light responses after 3 hours of hypoxia (Fig. 8).

## DISCUSSION

Despite extensive animal research, more than 90% of clinical trials for investigational new drugs fail to progress to market approval (*43*, *44*), often due to inadequate safety and efficacy data from animal models (*28*, *45*–*48*). Small-animal models and in vitro assays frequently struggle to replicate the complex pathologies of retinal diseases, e.g., age-related macular degeneration and glaucoma, which are influenced by advanced age, multiple genetic risk factors of low penetrance, and environmental and lifestyle factors (*28*). Nonhuman primates share key retinal features with humans, but current disease models in these species are limited to certain forms of inherited retinal degeneration (*49*), and their use is constrained by ethical concerns and high costs (*28*).

Research in human postmortem eyes, especially those from donors with retinal diseases, holds immense potential to overcome these limitations. Such research could elucidate disease mechanisms and facilitate the development of new treatments, offering hope to millions of patients who face vision loss and diminished quality of life. Several challenges have previously restricted the use of human postmortem eyes for large-scale physiological experiments, including the need for ultrafresh human tissue, the difficulty of preserving light responses in the laboratory, and challenges in sampling precise retina locations.

We address these barriers by establishing a robust infrastructure in collaboration with the Utah Lions Eye Bank and DonorConnect, enabling weekly recovery and transportation of light-responsive human eyes from DBD and DCD donors (Figs. 1 and 3). By scaling access to functionally viable human tissue, we lay the foundation for detailed studies of human retinal physiology, neural network activity, and pharmacological testing.

In our previously published data, mouse and human photoreceptors more effectively maintained their light responses during postmortem ischemia and better recovered their function immediately after hypoxic injury compared to ON-bipolar cells (*6*). Here, we report that ON-bipolar cell light responses, particularly in the peripheral retina, can be restored by overnight incubation of eyecups in oxygenated Ames’ media, even after 60 min of postmortem ischemia or hypoxia (Figs. 1 and 5). Peripheral rod ON-bipolar cell responses were sometimes even restored in research donor eyes enucleated 3 to 4 hours postmortem, although their responses remained smaller than those recorded from freshly recovered organ donor eyes, and photoreceptor light response amplitudes consistently improved (fig. S5).

Previous studies have shown that while the structural organization and spontaneous activity of RGCs can be preserved in in vitro long-term cultured retinal patches (*23*), their light responsiveness declines with prolonged culture or ischemia, indicating that functional impairment precedes cell death. In this study, we preserved light responses in intact posterior human eyes for up to 48 hours postmortem (Fig. 1), contingent upon retinal attachment to the RPE and the absence of microbial contamination. However, light responses from central retinal ON-bipolar cells initially remained elusive. Our observations suggest that this may, at least in part, be explained by the higher risk of retinal detachment in the central retina of enucleated eyes; however, in our most recent experiments, we have been able to preserve light signal transmission also in the central human retina at least up to 24 hours (Fig. 3, H to J). In some cases, inner retina signals were compromised, while in other cases, they were even stronger after 24 hours than in fresh controls. These data suggest that, similar to the peripheral retina, the central retina can heal and maintain light responsiveness after the initial ischemic injury when an optimal environment and intact posterior eye structure are preserved.

While postmortem ischemia remains the strongest predictor of retinal function (*6*), variability between donors is likely influenced by additional factors, including medical conditions, undocumented periods of ischemia or systemic stress before life support, and compromised ocular perfusion during brain death and/or life support. Our ethical approval currently does not permit us to assess light responses before enucleation, limiting our ability to prospectively identify high-quality human donor eyes. Furthermore, even partial retinal detachment during incubation likely reduces or abolishes light responses. These challenges are unique to working with human donor eyes and highlight the need for more comprehensive clinical documentation and refined recovery protocols.

Although our primary focus was on light responses in photoreceptors and ON-bipolar cells, we also detected light-evoked activity in RGCs after 70 min of enucleation delay and 24 hours of posterior eye incubation (Fig. 1, J and K, and fig. S3). Our analysis distinguished ON-, OFF-, and ON/OFF-RGCs. However, it remains unclear how these responses compare to those in fresh eyes, and at which point inevitable axotomy during enucleation leads to loss of function and RGC death.

Our hypoxia model directly controls tissue oxygenation by bypassing the blood-retina barrier and most closely models postmortem ischemia. However, ischemia-reperfusion injury in vivo involves additional processes beyond oxygen deprivation and reoxygenation, including breakdown of the blood-retina barrier, vascular leakage, and infiltration of circulating immune cells into the neural retina. These inflammatory and vascular components are not replicated by our ex vivo system, which instead isolates the neural aspects of ischemic damage and recovery. Therefore, our findings should be interpreted within this context.

The retinal circulation (which supplies the inner retina, including the synaptic connections, ON-bipolar and RGCs) fails more commonly in disease than the choroidal circulation (which supplies the more hypoxia-resilient photoreceptors). Choroidal blood flow is typically preserved, except in cases of CRAO with choroidal involvement or ophthalmic artery occlusion. Given the greater vulnerability of ON-bipolar cells to hypoxia, coupled with the higher risk of losing retinal circulation, dysfunction of the inner retina, particularly of ON-bipolar cells, may represent a key bottleneck in signal transmission after hypoxia. As such, we speculate that ON-bipolar cells should be a central target for neuroprotective strategies.

The greater susceptibility of ON-bipolar cells to hypoxia, compared to photoreceptors, may stem from their retinal microenvironment: ON-bipolar cells reside in the inner retina, which is directly supplied by the retinal vasculature; photoreceptors occupy the avascular outer retina, which is mostly supplied by the choroidal vasculature. The vast amounts of oxygen consumed by photoreceptors result in a rapid drop to extremely low oxygen concentrations, particularly in their inner segments and in the dark (*50*). As a result, photoreceptors function under conditions that would constitute hypoxia or anoxia for many other cell types. The apparent vulnerability of ON-bipolar cells could, therefore, be rephrased: as the remarkable resilience of photoreceptors to hypoxia.

Our findings challenge the widely held notion that ischemic or hypoxic injury irreversibly damages neurons, including those in the retina. Instead, they suggest that substantial neuronal recovery is possible with timely restoration of perfusion (Figs. 1 and 5). This aligns with a recent study reporting cellular recovery in pig brains following 1 hour of warm ischemia (*5*). Our data show that, instead of collectively undergoing hypoxia-induced apoptosis, a sizeable proportion of retinal neurons survive (Fig. 6). Temporary loss of ON-bipolar cell light responses must, therefore, be attributed to loss of synaptic input from photoreceptors and/or ischemia-induced membrane potential dysregulation, preventing light signal transmission in ON-bipolar cells.

However, irrespective of the mechanism(s) underlying the transient loss of ON-bipolar cell function, we importantly not only extend access to functionally viable human eyes but also question whether ischemic or hypoxic retinal or brain injury is truly irreversible and can be repaired as long as cells are reperfused within a critical time frame. Even though we consistently restored and preserved cone photoreceptor light responses in the maculae from DCD and research donors, and rod-mediated photoreceptor and ON-bipolar cell light responses in the peripheral retina, signal transmission to cone ON-bipolar cells remained elusive, indicating that the central retina and/or cone pathway may be more susceptible to ischemic injury. Our future research should, therefore, identify improved methods that consistently retain or restore ON-bipolar cell light responses in the central retina.

Ex vivo ERG in human donor retinas overcomes many disadvantages of alternative techniques, particularly when studying age-related physiological changes and multifactorial diseases. Unlike mice with their shorter lifespans and retinal organoids, which largely replicate retinal development (*17*, *28*), our platform provides access to mature human tissue. Nonhuman primates, while anatomically similar to the human retina, including a macula, lack relevant disease models and face limitations due to high costs and ethical concerns. Our approach provides a practical, human system to study central vision loss, which cannot be effectively studied in nonprimate mammals.

Our study provides protocols and technologies for long-term functional preservation of eyecups, precise sample collection, mechanistic studies, and drug testing (Figs. 1 to 5). Using these methods, we investigated pathways involved in ischemia-reperfusion injury in the retina, with a focus on oxidative stress and glutamate excitotoxicity (Figs. 7 and 8). Our experiments highlighted that targeted inhibition of specific ROS-producing pathways, namely, succinate dehydrogenase (complex II) and NADPH oxidase, can partially protect photoreceptor function during and after hypoxia (Fig. 7). However, the protective effects were not additive or synergistic: Combining inhibitors of complex II and NADPH oxidase assembly yielded less protection than expected (fig. S8), and broad-spectrum ROS scavenging with a high concentration of α-lipoic acid was detrimental (fig. S8). This raises the possibility that low levels of ROS, possibly depending on their molecular identity, concentrations, or subcellular localization, may serve protective or signaling roles in retinal cells in the healthy retina and during repair after hypoxia, but that excessive ROS production is detrimental to photoreceptors.

We acknowledge that the current drug study does not yet fully clarify the underlying mechanisms of hypoxic damage and repair, these clearly warrant further investigation. Future work should particularly focus on our intriguing observation that inhibiting ROS production was beneficial to photoreceptors but not to ON-bipolar cells. Additional work should also directly measure ROS, which may correlate with drug effects on light responses in specific retinal cell types. Additional dose-response and time-course experiments could shed light on the relationships between drugs, ROS modulation, and retinal function.

While limiting ROS production improved photoreceptor function, ON-bipolar cell responses did not show similar recovery (Fig. 7), indicating that different retinal cell types may require distinct protective strategies. The unchanged ON-bipolar cell output, despite increased photoreceptor input, suggests that ROS signaling may play a role in maintaining ON-bipolar cell responses, rather than merely causing damage. We tested a limited number of pharmacological compounds and focused on ROS production as a mechanism of damage. Nevertheless, our results demonstrated the usefulness of the ex vivo ERG platform: to evaluate functional outcomes of pharmacological interventions in the retina and as a model of the CNS.

As a key limitation of our study, many mechanistic questions remain unresolved regarding the nature and extent of cellular damage and recovery. For example, we did not determine whether hypoxic damage occurs primarily at the photoreceptor–to–ON-bipolar cell synapses or within the ON-bipolar cells themselves, nor identified the precise cellular and molecular mechanisms of damage, which could involve disrupted synaptic signaling, mitochondrial dysfunction, plasma membrane depolarization, or loss of intracellular calcium homeostasis. While light-evoked photoreceptor and ON-bipolar cell responses recovered after hypoxic damage, it remains unclear whether this reflects a full functional recovery, with intact synaptic integrity, mitochondrial function, energy metabolism, and calcium homeostasis, or residual electrophysiological activity in structurally or metabolically compromised cells. While outside the scope of our present work, future studies should address these questions, as well as whether retinal cells that regain function after hypoxic and ischemic insults also survive in the long term.

Our findings also have implications for developing treatments for acute retinal ischemia, e.g., in CRAO. Current treatments, including hyperbaric oxygen therapies and intraocular pressure reduction, are often insufficient, and patient care lacks effective treatment options. Our ex vivo data suggest that supplying the retina with oxygen within a critical time frame of less than 3 hours can markedly benefit light responses in retinal neurons. This raises the possibility that vitrectomy and perfusion of the vitreous cavity with oxygenated media, possibly in combination with ROS-targeting or neuroprotective drugs, could be developed as a therapeutic intervention for CRAO to mitigate ischemia-reperfusion injury until retinal perfusion is restored.

Our study may even extend beyond ophthalmology, potentially offering insights into ischemia-reperfusion injury in other CNS tissues. The retinal ischemia-reperfusion model may inform treatment strategies for acute damage following ischemic stroke or brain injury after cardiac arrest. Our ex vivo platform is designed to bridge the gap between in vitro assays and clinical testing by enabling functional testing in the intact human retina. Our approach provides human-specific information that may more accurately predict clinical outcomes for investigational new drugs. Although we have not yet correlated in vivo and ex vivo light responses in donor eyes, our work lays the foundation for such comparisons in future studies. Certain aspects, such as ex situ perfusion of human eyes, will be critical for improving physiological function and translational relevance.

Last, our work will support efforts to replace retinas or even whole eyes, similar to the pioneering whole-eye transplantation recently performed at NYU Langone Health (*51*, *52*) and the audacious program announced by the Advanced Research Projects Agency for Health (ARPA-H) to restore vision in bilaterally blind patients through Transplantation of Human Eye Allografts (*53*). Our methods for recovering, restoring, and preserving light responses in human donor eyes are directly applicable to these efforts by supporting light responses in the central and peripheral retina from DBD and DCD organ donors.

## MATERIALS AND METHODS

### Donor demographics, eye procurement, and transportation

Human eye tissue was procured by the Utah Lion’s Eye Bank (Food and Drug Administration accreditation number 3000215349) in conjunction with whole organ donation by Donor Connect (Food and Drug Administration accreditation number 3000215350, Centers for Medicare & Medicaid Services accreditation number 46P001). The Utah Lion’s Eye Bank obtained informed consent for research use for all donor eyes according to the Uniform Anatomical Gift Act (UAGA). The research team did not identify or select potential organ donors and had no access to nonanonymized donor information, except for information on ocular diseases, such as age-related macular degeneration and diabetic retinopathy. For this study we exclusively used donors without known retinal diseases. Our research project was, therefore, classified as exempt by the University of Utah (IRB no. 00106658).

Whole eyes or posterior poles were enucleated within 30 to 60 min after cross-clamp and transported to the laboratory in HEPES-buffered Ames’ media (A1420, Millipore Sigma) that was directly bubbled with 100% O_2_ at room temperature in the custom transportation case we previously described (*6*).

Eyes from research donors were enucleated up to 5 hours postmortem by a trained Eye Bank technician and immediately placed into HEPES-buffered Ames’ media at room temperature. Eyes from organ donors were obtained up to 1 hour after cross-clamp at the time of organ removal, either with or without corneas, if these were transplanted, and placed into HEPES-buffered Ames’ media and oxygenated with 100% medical oxygen in our custom transportation case. From the time of retrieval, eyes were protected from light, stored at room temperature, and immediately transferred to the laboratory. Table S1 lists basic nonidentifying information: age, sex, cause of death, cross-clamp/death-to-preservation delay, and potentially relevant medical conditions of all organ and research donors used in this study.

### Sample preparation

Upon arrival in the laboratory, eyes were handled under dim red-light illumination and stored in bicarbonate-buffered Ames’ media oxygenated with 95% O_2_ and 5% CO_2_. By making an incision approximately 3 to 5 mm behind the limbus, we removed the anterior section of the eye with the lens, if applicable, and removed most of the vitreous to image the retina.

### Hardware

#### OCT adaptation and sample acquisition

We vertically reoriented an OCT machine (Spectralis, Heidelberg Engineering, Franklin, MA) to ensure that dissected eyecups would remain in place during imaging and sampling. With Fusion 360 (Autodesk, San Rafael, CA), we machined an adapter plate from 6061 aluminum and secured the OCT scanner to additional mounting equipment (ThorLabs, Newton, NJ) uprightly. This and all other custom-made models and mechanical drawings are available at Zenodo (https://zenodo.org/records/16014189).

We secured the eyecup in a custom-made vacuum chamber, which we had modeled in Fusion 360 and machined in 6061 aluminum. When we applied pressure of 1.773 × 10^4^ Pa to porcine eyecups, they remained firmly in position with no discernable signs of external stress. Our custom fixture stage included a LX20/M translational state (X ± 25 mm and Y ± 25 mm), VAP4/M vertical translation stage (Z ± 101.6 mm) and TTR001/M tip, tilt, and rotational stage (roll α ±5°, pitch β ±5°, and yaw γ ±10°, ThorLabs, Newton, NJ), which provided six degrees of freedom to position the eyecup precisely. After locating the fovea with OCT, we punched a 5-mm retina sample with a disposable biopsy punch (Thermo Fisher Scientific, Hampton, NH) on a 3D-printed lens attachment. Elastic O-rings (McMaster-Carr, Elmhurst, IL) reliably and safely secured the sliding biopsy mechanism to the OCT lens.

#### Positional accuracy validation

To validate the precision of the punch attachment, we marked 24 circles (~3 mm in diameter) as targets onto an affixed sticky note (3M, St. Paul, MN). A needle puncture outside the targets was marked with tape as the “reference area” before each target was translated to this location and punctured. The distance from the center of each hole to the target was recorded and reported as the mean difference and SD. We used porcine eyecups to test the system’s ability to excise retina samples accurately. To prepare the eyes for testing, we made a small incision near the limbus, submerged the eyes overnight in 4% paraformaldehyde in phosphate-buffered saline, and removed the anterior segment and most of the vitreous.

Using a 2-mm disposable biopsy punch, we made a minor impression in the retina to serve as a reference marker, whose location we marked on the OCT display to compensate for the offset between the OCT’s optical axis and the axis of the biopsy punch. We then aligned the target area with the calibration marker and penetrated the eyecup with the biopsy punch to the sclera. The procedure was deemed successful if the target was accurately incised. This process was repeated three times in three different eyes, resulting in nine data points (*n* = 9).

### Software

We combined several Python scripts to automatically collect retinal images and apply deep learning models that classify images and detect objects. The main program, auto_sample.py, relies on capture_coordinates.py, ImgClassRevised.py, train_obj_det.py, and test_obj_det.py scripts.

#### Screen coordinate capture

In the automated workflow of auto_sample.py, the capture_coordinates.py script allows the precise capture of spatial data to interact with the OCT software by interactively collecting screen coordinates and color information through mouse clicks. These coordinates detect when the OCT software is ready for image acquisition and execute a sequence of mouse clicks that will save the acquired images for further analysis. Upon execution, the user is prompted to specify the number of coordinates to be saved. By using both pyautogui (version 0.9.54), the program listens for mouse click events. For each click, it captures and stores the corresponding screen location and the color at that point, leveraging pyautogui.screenshot().getpixel() to obtain the color information. This process documents the selected regions, allowing for automated mouse movement in auto_sample.py.

#### Image classification

We trained the classification model in auto_sample.py using ImgClassRevised.py to sort OCT images into two categories, either with or without a fovea. The script initially sets up the environment and imports necessary libraries, including TensorFlow (version 2.10.1), to build and train the neural network and OpenCV (version 4.12.0.88) for image manipulation. TensorFlow’s image_dataset_from_directory function loads and labels “fovea” and “no fovea” images from predefined directories based on their folder structure, resizes the images to reduce the computational power needed to train the model, and places the images into batches of 12. The classification model was trained on 78 OCT images from human donor eyes that we had acquired under the same protocol before AI integration, evenly split between fovea or no fovea categories.

Most of the script preprocesses images by applying random brightness, contrast, saturation, and hue adjustments to optimize the model. The model’s architecture is constructed using TensorFlow’s Keras API (version 2.10.0), starting with a convolutional base comprising four convolutional blocks, each followed by max pooling to distill features from OCT images efficiently. The initial block introduces 32 3 by 3 filters with ReLU activation, progressively increasing the filter count to 64 in the second, 128 in the third, and 256 in the fourth to capture a wide array of image features. After flattening the output to a single dimension, the model integrates two dense layers with 512 and 256 neurons, respectively, using ReLU activation to process the features further. In the final step, a single neuron with a sigmoid activation function designed for binary classification discerns images from those with or without a fovea.

The dataset was divided into training, validation, and test sets to evaluate the model’s performance accurately. Callbacks in the script save the best-performing model with the highest validation accuracy and log training progress, thus facilitating model tuning and evaluation. After training, the script plots loss and accuracy metrics over 30 epochs to visualize the learning process. Last, users can save the trained model for future use.

#### Object detection

The train_obj_det.py script delineates the training process of our object detection model and allows auto_sample.py to detect the location of the fovea in classified OCT images with the latest YOLOv8 architecture. The script uses the Ultralytics YOLO library (version 8.3.167) to train a YOLO model based on a predefined configuration file (yolov8n.yaml), which points to a separate training dataset of 292 annotated foveal images, consisting of donor and OCTDL open source data from normal eyes (*54*). The train_obj_det.py script ensures the use of GPU if available for faster processing. Its core function, locate_fovea, takes an image path and the YOLO model as inputs and runs inference on the image to predict the presence of a fovea and plots and displays its location. A matplotlib library (version 3.10.3) visualizes the results and uses the PIL library (version 11.0.0) for image processing. After training over 30 epochs, the script validates the model’s performance using a separate set of 39 foveal images.

#### Automatic sampling

With the data and models from the other scripts, auto_sample.py automatically locates and samples the foveal region of the retina. It initially monitors a predefined screen region to match a target color, indicating that the operator has obtained several b-scans of the retina, and saves the images from the OCT software. The images are each assigned a unique identification number to avoid overwriting existing data, which enables easy access for subsequent processing. The pretrained script iterates through the saved images, applies necessary preprocessing, and classifies images that contain a fovea. Although the script may find some false-positive images in the peripheral retina, it retains only the longest sequence of positive images, thus indicating the true location of the fovea. The central image of this sequence is kept, as it provides the best representation of the central foveal region.

To obtain the coordinates of the fovea relative to the reference area recorded earlier, the script precisely locates the green b-scan line as the *y* coordinate through color space conversion, masking, and edge detection techniques, followed by a Hough line transform. Since the scan line on the fundus image is a 1:1 match for the adjacent OCT image, the program can gather the *x* coordinate of the fovea based on its coordinates in the OCT image. The script first calibrates the X and Y stepper motors via the Telemetrix library (version 1.4.3) by identifying the difference in the fovea’s location after moving the stage a set distance. Subsequently, it moves the motors to position the fovea directly underneath the biopsy punch for sample acquisition.

### Sample handling, preservation, and ex vivo ERG

Trained Utah Lion’s Eye Bank technicians recovered human donor eyes, which they immediately submersed in HEPES-buffered Ames’s media (Sigma-Aldrich, St. Louis, MO), protected from light, and transferred to the laboratory (*6*). In the laboratory, the tissue was processed under dim red-light illumination, and the donor eyes were immediately transferred into bicarbonate-buffered Ames’ media, which was oxygenated with 95% O_2_ and 5% CO_2_. The anterior segment and most of the vitreous was removed, and the retina was imaged using infrared light and OCT (Spectralis, Heidelberg Engineering, MA). To store eyecups or retinal punches attached to the underlying RPE/choroid until ex vivo ERG or for long-term incubation to recover light responses following postmortem delay to enucleation, human eyecups and punches were placed in a deep glass dish filled with bicarbonate-buffered Ames’ media, which was directly bubbled with 95% O_2_ and 5% CO_2_ in darkness.

Wild-type C57BL/6J mice (7- to 19-week-old males and females) and *Gnat2* knockout mice (*55*) (3- to 7-month-old males and females) were euthanized with CO_2_ asphyxia followed by cervical dislocation, with approval from the Institutional Animal Care and Use Committee at the University of Utah (protocol number 1656). Eyes were immediately enucleated and dissected by piercing the cornea with a 30-gauge needle, removing the cornea and iris while leaving the lens in place to ensure we did not disrupt the close interaction between the retina and underlying RPE. We stored mouse eyecups in a 100-ml Pyrex bottle filled with Ames’ media, which we either exposed to 95% N_2_ and 5% CO_2_ to induce hypoxia or oxygenated with 95% O_2_ and 5% CO_2_; drugs were directly added to the Ames’ media. Retinas were dissected after 1 hour of hypoxia or after 3 hours of hypoxia followed by overnight recovery in oxygenated Ames’ media, with or without drugs.

Human retina samples were obtained as previously described, placed in the specimen holder, and initially perfused with oxygenated bicarbonate-buffered Ames’ media (bubbled with 95% O_2_ and 5% CO_2_) for 20 min. The perfusion solution was then switched to bicarbonate-buffered Ames’ media bubbled with 95% N_2_ and 5% CO_2_ to induce hypoxia for 1 hour, after which light responses were recorded. Oxygenated media was then reintroduced for 15 min to allow the retina to recover, after which restoration of light responses was assessed.

### ERG recording protocol

Ex vivo ERG (*56*) was recorded as described previously (*6*, *57*). Human or mouse retinas were carefully removed from the underlying RPE/choroid and mounted on the ex vivo ERG specimen holder, as described previously. The retinas were superfused with Ames’ media containing 100 mM BaCl_2_ to block K^+^ channels on Müller glial cell with or without 40 mM DL-AP4, which blocks signal transmission between photoreceptors and ON-bipolar cells (*30*, *58*). This allowed the recording of isolated photoreceptor light responses and combined photoreceptor and ON-bipolar cell responses, respectively, and the calculation of isolated ON-bipolar cell responses. If eyecups had been incubated with drugs during 3 hours of hypoxia and/or overnight recovery, we provided the same drug and concentration in the superfusate, except for DMM (a blocker of complex II in the mitochondrial electron transport chain) and MK-801 (an NMDA receptor blocker). We plotted photoreceptor and ON-bipolar cell amplitudes as a function of light flash strengths (photons μm^−2^) and displayed arithmetic means ± SEM.

### Multi-electrode array recordings

After 24 hours of incubation of a human donor eyecup in oxygenated Ames’ media, a 2 by 2 mm peripheral retinal punch was mounted RGC-side down on a 60-channel MEA (MEA 2100, Multi Channel Systems MCS GmbH, Reutlingen, Germany) and continuously perfused with oxygenated Ames’ media. Electrodes (30 μm diameter and 200 μm spacing) recorded RGC activity, with the tissue secured by a dialysis membrane (Thermo Fisher Scientific, Hampton, NH) attached to a stainless-steel washer. Recordings were acquired with Multi Channel Experimenter software (Multi Channel Systems MCS GmbH, Reutlingen, Germany) and digitized at 25 kHz. Signals were high-pass filtered at 300 Hz using a two-pole Butterworth filter, and spikes were detected with a threshold of five SDs below the mean background noise. Spikes were sorted using Offline Sorter (Plexon, Dallas, TX), and data were plotted with OriginPro (OriginLab Corporation, Northampton, MA, USA) and custom Python scripts.

### Small-volume closed perfusion system

We built a closed perfusion ex vivo ERG system with a peristaltic pump to circulate small volumes of perfusate. For these experiments, we used pieces of peripheral retina from human donor eyes with enucleation delays of one (UFT8, see table S1) to 4 hours (D54_2 and D55, see table S1), which had been incubated overnight. Rod photoreceptor responses were recorded at multiple time points over a 12-hour period. The perfusate consisted of HEPES-buffered Ames’ media at 32.0° ± 0.5°C with penicillin-streptomycin (Sigma-Aldrich), which was passed through a 1-μm filter, to prevent bacterial contamination. To isolate the photoreceptor component of the ERG response, 100 μM BaCl_2_ and 40 μM DL-AP4 were added. The dark-adapted retina was stimulated with 5-ms flashes at 505 nm. Two dripping burettes were used to break the electrical connection and dampen mechanical pulsations from the peristaltic pump. The perfusion system was grounded with Ag/AgCl electrodes (model EP2, World Precision Instruments) through agar gel bridges within the Faraday cage.

### TUNEL and IHC staining

Eyecups from *Gnat2*^−/−^ mice were incubated in either hypoxic or oxygenated Ames’ media, and cryoprotected in a sucrose gradient of 10 to 30%, embedded in 20% sucrose in OCT, and cryosectioned. Ten-micrometer retinal sections were blocked with 10% normal goat serum, 0.1% Triton X-100 in phosphate buffered saline (blocking buffer), and stained using Click-iT TUNEL kit (Thermo Fisher Scientific catalog no. C10617), mouse anti-PKCα H-7 (1:500, Santa Cruz Biotechnology catalog no. 8393, RRID: AB_628142), rabbit anti-cone arrestin (1:1000, Sigma-Aldrich Chemicon catalog no. 15282, RRID: NA), rabbit anti-Iba1 (1:200, Fujifilm Wako catalog no. 019-19741, RRID: AB_839504), rabbit anti-Iba1 (1:200, Abcam catalog no. 178847, RRID: AB_2832244), mouse anti-GFAP (1:200, Abcam catalog no. G3893, RRIB: AB_477010), and the nuclear stains 4′,6-diamidino-2-phenylindole (DAPI) (DAPI Fluoromount-G, Southern Biotech, catalog no. 0100-20) and Hoechst (1:10000, Invitrogen catalog no. H3570). Secondary antibodies included goat anti-mouse Alexa Fluor 555 (Invitrogen catalog no. A-21422, RRID: AB_141596) and goat anti-rabbit Alexa Fluor 647 (Invitrogen catalog no. A-21245, RRID: AB_141775) used at a dilution of 1:1000. Imaging was performed using a confocal microscope (LSM Airy Scan, ZEISS). Sections were selected on the basis of intact morphology identified by DAPI staining. Two independent, blinded researchers analyzed the images using ImageJ. Cell counts were averaged between both researchers, and all cell counts were normalized to the total number of PKCα-positive cells in the inner nuclear layer or DAPI-positive cells in the photoreceptor cell layer.

### Statistical analysis

Statistical analysis was performed by two-way analysis of variance (ANOVA) followed by Bonferroni post hoc test to compare experimental groups across multiple light intensities. For comparisons between two groups, unpaired *t* test was used. Significance levels were defined as follows: *P < 0.05, **P < 0.01, and ***P < 0.001 (versus oxygenated control), and ‡‡*P* < 0.01 and ‡‡‡*P* < 0.001 (versus hypoxia).
