# Supplementary material for: Healing of ischemic injury in the retina
Source: Sci Adv. 2026 Jan 21;12(4):eadx7204. doi: 10.1126/sciadv.adx7204 (PMC12822649; doi:10.1126/sciadv.adx7204)
Supplement: Supplementary file 1 — Figs. S1 to S8 Table S1 [file sciadv.adx7204_sm.pdf]

Science Advances
AAAS

Supplementary Materials for

Healing of ischemic injury in the retina

Silke Becker *et al.*

Corresponding author: Frans Vinberg, frans.vinberg@utah.edu, frans.vinberg@uci.edu

Sci. Adv. **12**, eadx7204 (2026)
DOI: 10.1126/sciadv.adx7204

This PDF file includes:

Figs. S1 to S8
Table S1

Fig. S1.

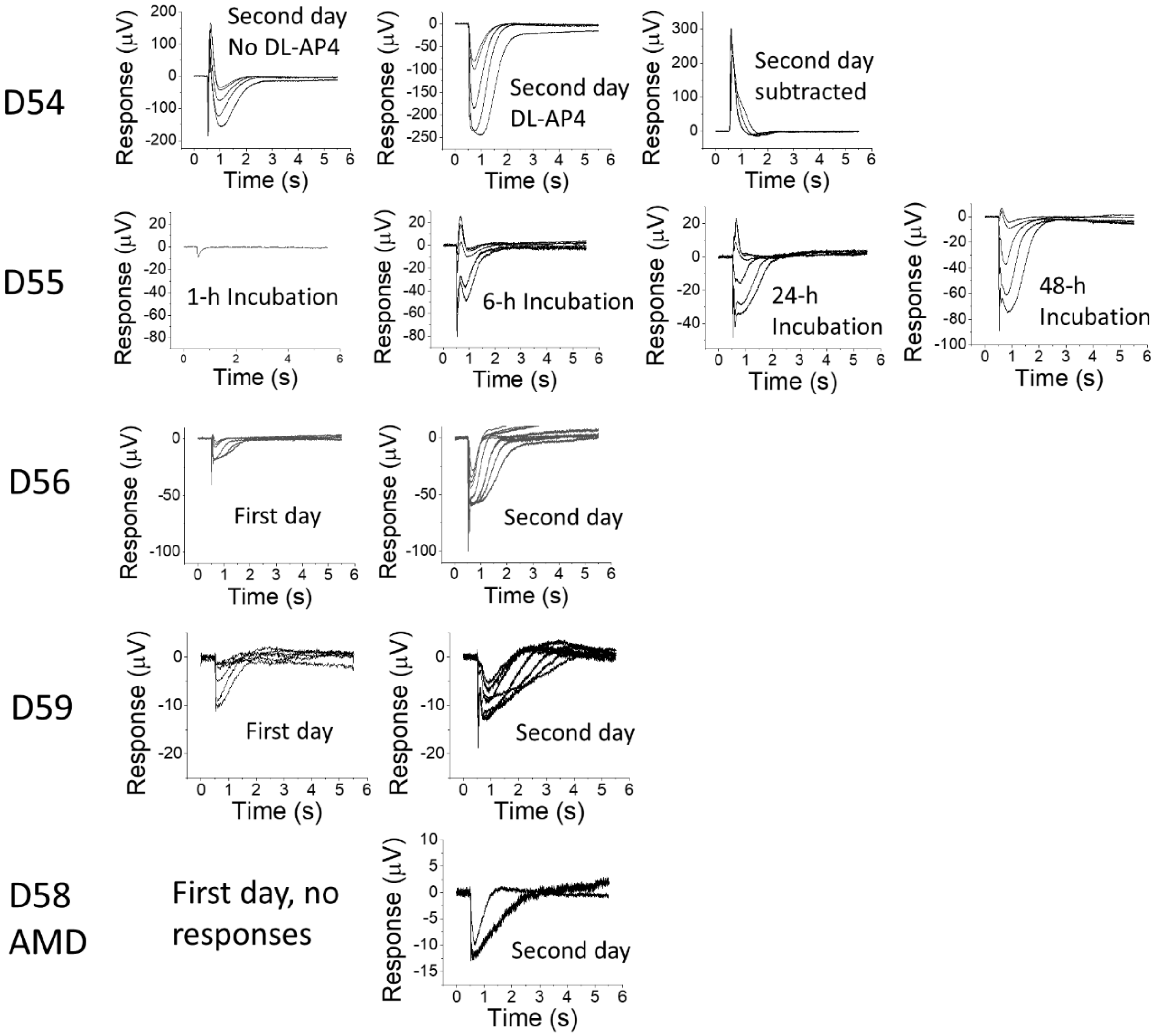

Impact of overnight eyecup incubation on the light responses in research donor eyes enucleated 3-4 hours postmortem. Light responses from peripheral retina samples from five research donors (D54, D55, D56, and D58_AMD) from whom eyes were recovered 3-4 hours postmortem (see Table S1 for donor details). D54 and D58_AMD recordings were conducted only after overnight incubation in oxygenated Ames'. Unless indicated otherwise, samples were perfused with Ames' media containing 100 µM Ba^{2+}. For D54, responses are shown in the absence (left) and presence (middle) of 40 µM DL-AP4. The rightmost panel for D54 shows responses derived by subtracting data in the middle panel from that in the left. Light flash strengths (green photons μm^{-2}): D54, 19 – 1,024; D55 (6-h, 24-h and 48-h): 19 – 2,050; D59, First day: 2,300 – 2,500,000, Second day: 19 – 4,100; D56, First day: 57 – 3,900; Second day: 57 – 7,800; D58_AMD: 19 – 16,400.

Fig. S2.

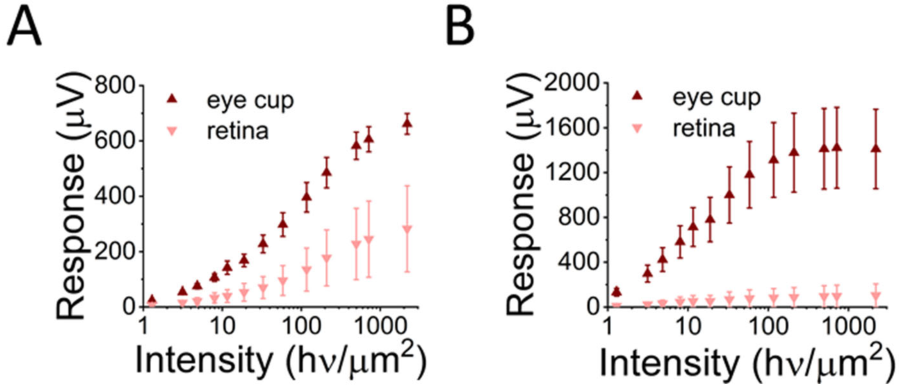

RPE dependence of retinal function after overnight storage. (A) *Ex vivo* ON-bipolar and (B) photoreceptor cell light responses after overnight storage of eye cups (N=4) or isolated retinas without underlying RPE (N=3).

Fig. S3.

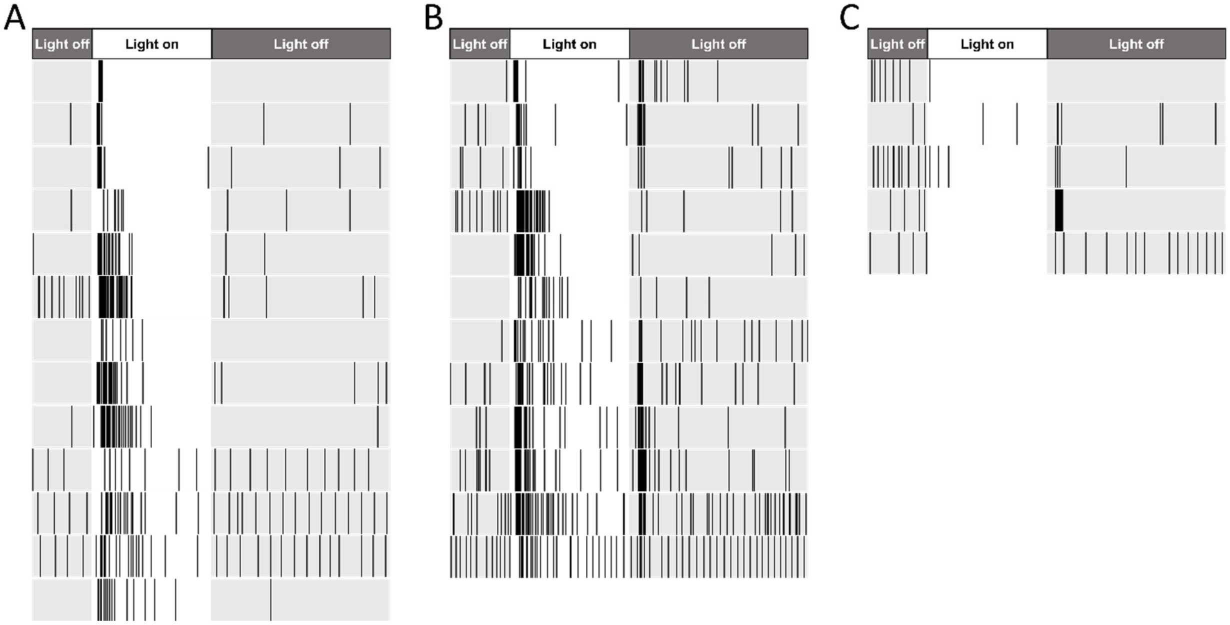

All retinal ganglion cell responses to 1-second flash of white light (24 cd/m^2) from donor ARPAH6 after 24-h enucleation. (A) ON-RGC responses from 13 cells. (B) ON/OFF RGC responses from 12 cells. (C) OFF-RGC responses from 5 cells.

Fig. S4.

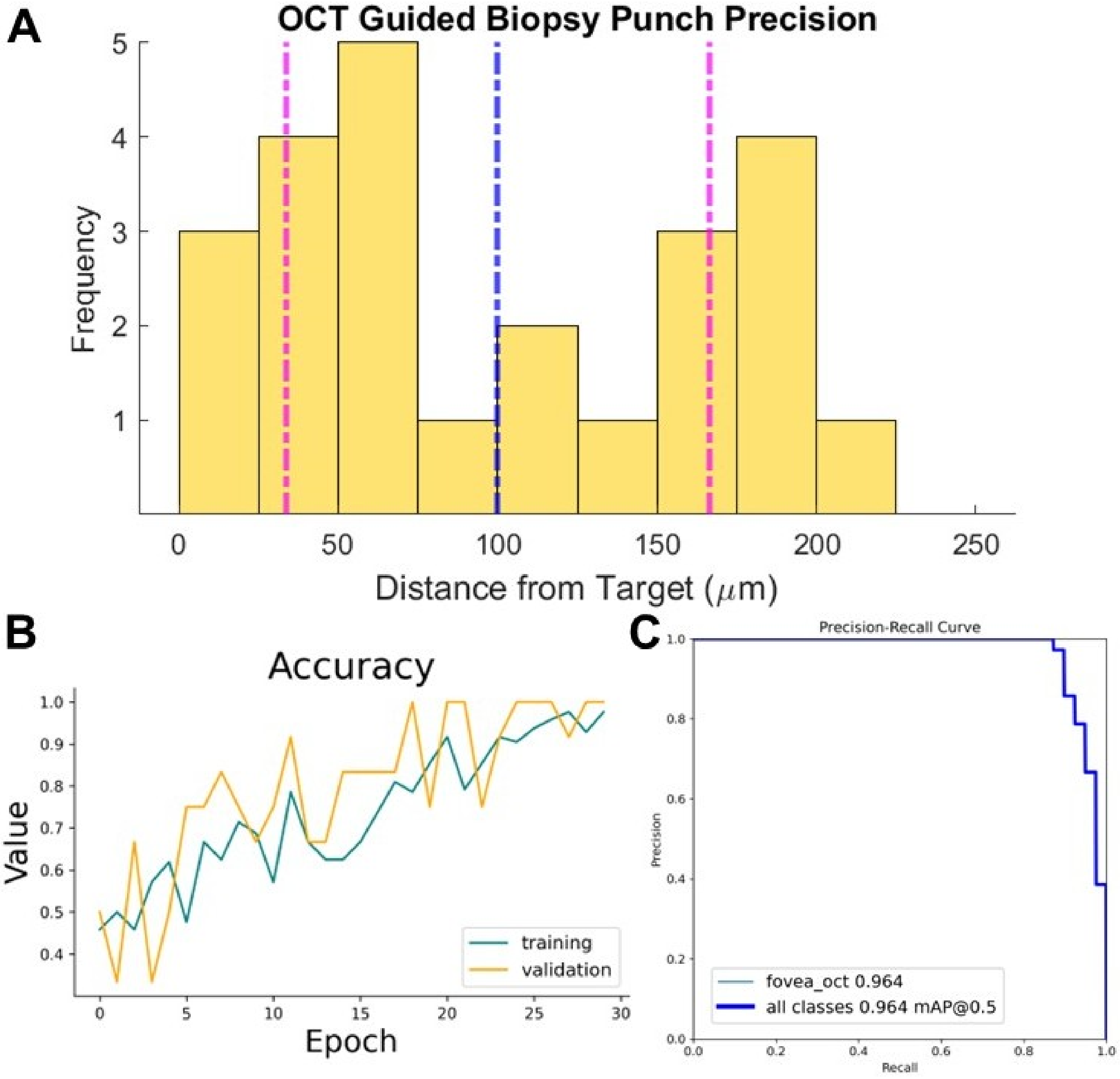

OCT and AI model accuracy and precision. (A) Bar chart of OCT precision test displaying the distances between each trial punch (N=24) from the target, with a mean (blue) of 100 ± 66.4 µm (purple). The range of each histogram bin is 25 µm. (B) Accuracy of classification model training on 78 images. The best checkpoint was used for the final model, where accuracy in training and validation was 98% and 100%, respectively. (C) Precision-Recall curve of object detection model trained on 292 images. The Mean Average Precision at 50% IoU threshold is 96.4%.

Fig. S5.

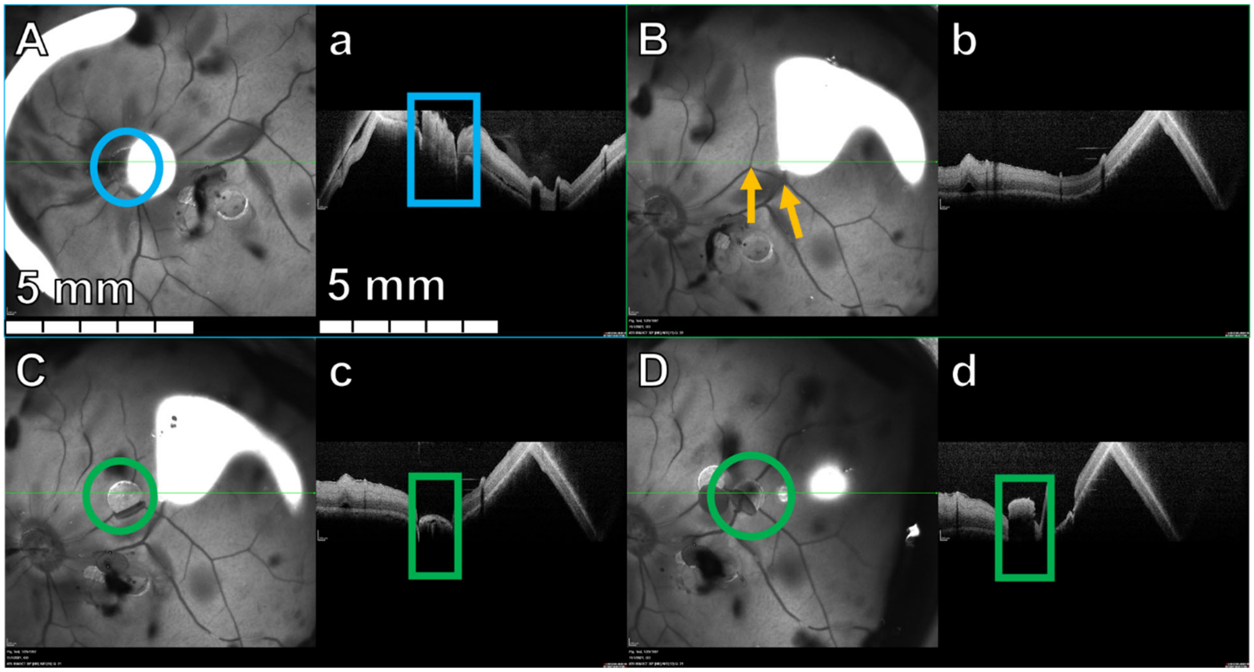

Sampling precision in porcine retina. Fundus images (capital letters) are captured with infrared imaging. OCT images (lowercase letters) show cross sectional analysis (relative to b-scan; green line). A reference punch is made (A,a; blue). Two targeted areas are selected (orange arrows in (B)). Biopsy punches are taken (green), (C,c), and (D,d). 100% of targeted areas were successfully sampled (n=9).

Fig. S6.

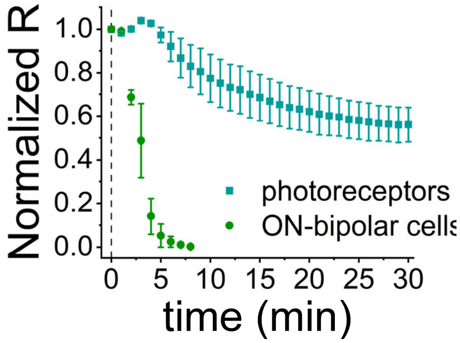

Sensitivity of retinal neurons to hypoxia. Photoreceptor (blue) and ON-bipolar cell responses (green) during hypoxia (2.5% O2), normalized to the response amplitudes before the onset of hypoxia (mean ± SEM, N=3 for photoreceptors, N=2 for ON-bipolar cells).

Fig. S7.

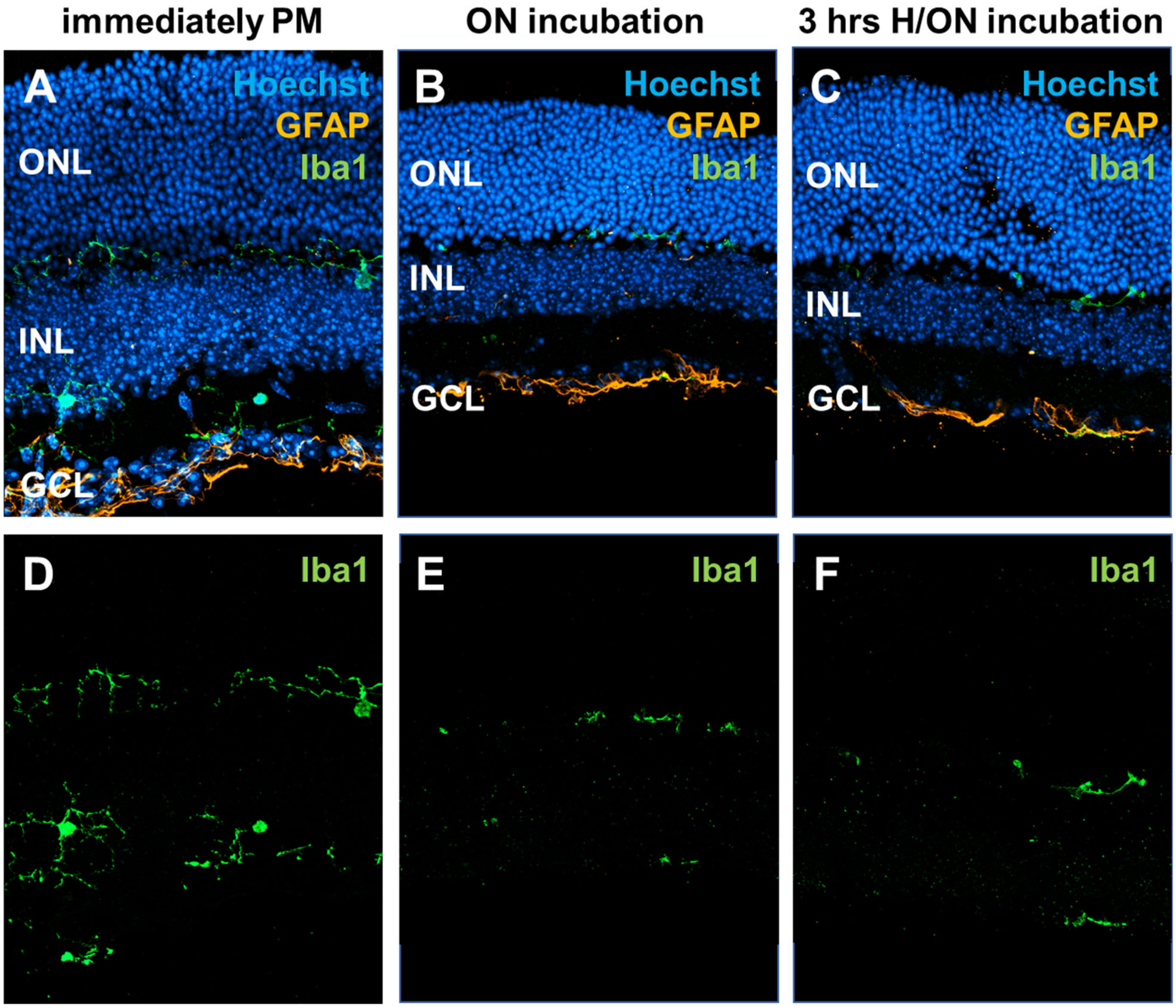

Müller glia and microglia in the postmortem retina. Glial fibrillary acidic protein (GFAP, orange) expression was consistent with expression in astrocytes, but not Müller glia, in mouse retinas immediately postmortem (PM, A), after overnight incubation in oxygenated media (B), and after three hours of hypoxia followed by overnight incubation in oxygenated media (C). Staining with Iba1 (green) showed the typical ramified morphology and localization in the inner and outer plexiform layers of resting microglia (D). Following overnight incubation in oxygenated media, microglia were still located in the inner and outer plexiform layers, but began to assume a more activated cell morphology (E). This appearance was maintained in retinas that had been stored for three hours in hypoxia followed by overnight incubation in oxygenated media (F).

Fig. S8.

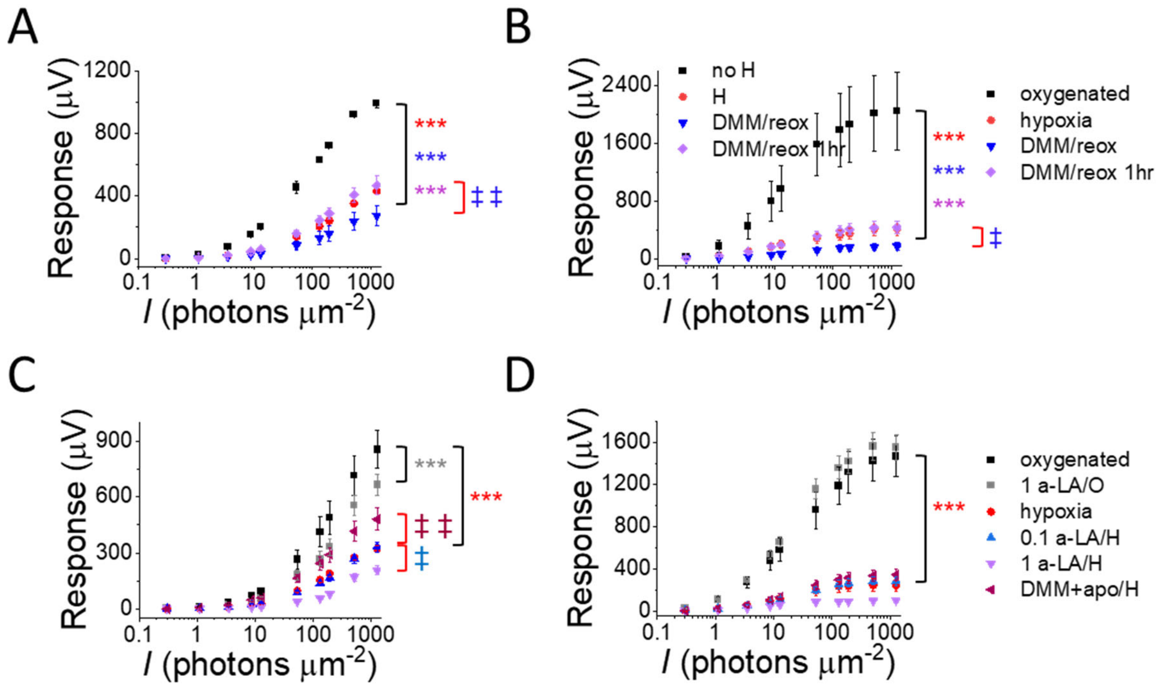

Antioxidant drugs during hypoxia and reoxygenation. Photoreceptor (A) and ON-bipolar cell light responses (B) after three hours of hypoxia with overnight incubation in oxygenated Ames' media in the absence or presence of dimethyl malonate (DMM) for 10 min before and one hour during reoxygenation or only during overnight reoxygenation (N=2-3). Photoreceptor (C) and ON-bipolar cell light responses (D) in oxygenated media or after three hours of hypoxia with overnight incubation in oxygenated Ames' media in the absence or presence of a-lipoic acid (a-LA) or DMM+apocynin (DMM+apo, N=4-5). Two-way ANOVA followed by Bonferroni post hoc test, *** $P<0.001$ (compared to oxygenated control), ‡ $P<0.05$, ‡‡ $P<0.01$ (compared to hypoxia).

Table S1.

**Donor ID**	**Donor type**	**Preservation delay/mins**	**Age/sex**	**Cause of death**	**Prior medical conditions**
UFT1 (only AI training/testing)	Organ donor	72	26/M	Trauma	COVID, cerebral edema, respiratory failure, lactic acidemia, C1 cervical fracture.
UFT2	Organ donor	54	56/male	SIGSW	Type 2 diabetes. no ocular.
UFT3	Organ donor	50	63/female	ICB/ICH	Astigmatism.
UFT5	Organ donor	30	64/male	Myocardial infarction	coronary artery disease, angina, atheroslerotic heart disease, afibrillation, cardiomypothy, high cholesterol, triple bypass, numerous cardiac conditions.
UFT6	Organ donor	33	37/female	Respiratory arrest	Asthma, morbid obesity, lactic acid increased. No ocular.
UFT7	Organ donor	22	54/female	ICB/ICH	Cardiomyopathy. No ocular.
UFT8 (Closed perfusion)	Organ donor	68	38/male	Anoxic brain injury	Cardiomyopathy, hypercholesterolemia. Glaucoma.
UFT10	Organ donor	77	57/female	ICB/ICH	Ischemic right carotid artery stroke
UFT11 (only AI training and testing)	Organ donor/NRP	211	62/F	ICB	non-traumatic intracerebral hemorrhage, hysterectomy
UFT12 (only AI training and testing)	Organ donor	65	46/F	Acute cardiac event	acute respiratory failure, cardiac arrest, elevated lactic acid, hypertension
UFT13 (only AI training and testing)	Organ donor	44	61/F	Orthopedic surgical complications	epilepsy, Raynaud's disease, neuropathy
ARPAH1	Organ donor	46	61/female	Cerebrovascular accident	LASIK.
ARPAH2	Organ donor	73	73/female	Acute respiratory failure leading to acquired brain injury	Type 2 diabetes, intraocular lenses in both eyes.
ARPAH4 (only AI training/testing)	Organ donor	24	57/M	Trauma	Type 2 diabetes, esophageal abnormality, gait instability, hypertension, peripheral neuropathy
ARPAH5 (only AI training/testing)	Organ donor	69	49/M	ICB/ICH	Hyperglycemia, Diabetes, pneumonitis.
ARPAH6	Organ donor	70	35/male	ICH	Surgical complications, ICH
ARPAH7	Organ donor	76	34/female	SAH	migraines, tobacco use, marijuana use
ARPAH9	Organ donor	40	54/male	CVA	ADHD, hypertension, obesity, OSA
ARPAH10	Organ donor	45	64/male	Not known	Not known
ARPAH11 (only AI training and testing)	Organ donor	74	25/M	SAH	HTN.
D42	Research donor	250	94/male	Pneumonia	Congestive heart failure, diabetes mellitus, diabetic eye disease
D53	Research donor	180	68/female	Cancer	Cardiomyopathy, sepsis
D55	Research donor	247	89/male		
D39 AMD	Research donor	173	90/female		AMD
D46 AMD	Research donor	346	75/male	Surgical complications	AMD, glaucoma
D48 AMD	Research donor	208	89/female	Acute Cardiac Event	
D51 AMD	Research donor	161	96/female	Myocardial infarction	AMD, glaucoma
D57 AMD	Research donor	219	93/female		
D54	Research donor	284	77/female	Cancer	Abnormal EKG, hyperlipidemia, hypertension

D54_2 (Closed perfusion)	Research donor	258	81/Male	Acute Cardiac Event	Chronic venous insufficiency, aortic stenosis, hypoxia, morbid obesity.
D55 (Closed perfusion)	Research donor	249	89/male	Not known	Not known
D59	Research donor	231	78/male	Sepsis	Stroke, lactic acidosis. Vision changes, vision loss.
D56	Research donor	232	82/female	Ischemic bowel	Liver cirrhosis, stroke, COPD, home O2 therapy.<br><br>Ocular stroke, visual field loss.
D58_AMD	Research donor	219	99/female	Pneumonia	AMD.
RD61	Research donor	259	88/female	Abdominal or thoracic aortic aneurysm	Eyelid OS drooping, AMD.

Donor information. Preservation delay: Time from cross-clamp (organ donors) or death (research donors) until preservation in Ames' media; NRP: Normothermic Regional Perfusion (arteries to the brain clamped); SIGSW: Self-inflicted Gunshot Wound; ICB/ICH: Intracranial Bleeding/Hemorrhage; COPD: Chronic Obstructive Pulmonary Disease; SAH: Subarachnoid Hemorrhage; CVA: Cerebrovascular Accident; OSA: Obstructive Sleep Apnea.
